# A high-throughput test enables specific detection of hepatocellular carcinoma

**DOI:** 10.1038/s41467-023-39055-7

**Published:** 2023-06-07

**Authors:** David Cheishvili, Chifat Wong, Mohammad Mahbubul Karim, Mohammad Golam Kibria, Nusrat Jahan, Pappu Chandra Das, Md. Abul Khair Yousuf, Md. Atikul Islam, Dulal Chandra Das, Sheikh Mohammad Noor-E-Alam, Moshe Szyf, Sarwar Alam, Wasif A. Khan, Mamun Al Mahtab

**Affiliations:** 1 HKG Epitherapeutics Ltd. Unit 313-315, 3/F Biotech Center 2, 11 Science Park west Avenue, Shatin, Hong Kong, SAR China; 2 Gerald Bronfman Department of Oncology, McGill University, Montreal, Canada; 3 International Centre for Diarrhoeal Disease Research, Bangladesh (ICDDR,B), Dhaka, Bangladesh; 4 Department of Hepatology, Bangabandhu Sheikh Mujib Medical University, Shahbag, Dhaka Bangladesh; 5 Department of Pharmacology and Therapeutics, McGill University, Montreal, Canada; 6 Department of Clinical Oncology, Bangabandhu Sheikh Mujib Medical University, Shahbag, Dhaka Bangladesh

**Keywords:** Diagnostic markers, Data processing, Tumour biomarkers

## Abstract

High-throughput tests for early cancer detection can revolutionize public health and reduce cancer morbidity and mortality. Here we show a DNA methylation signature for hepatocellular carcinoma (HCC) detection in liquid biopsies, distinct from normal tissues and blood profiles. We developed a classifier using four CpG sites, validated in TCGA HCC data. A single F12 gene CpG site effectively differentiates HCC samples from other blood samples, normal tissues, and non-HCC tumors in TCGA and GEO data repositories. The markers were validated in a separate plasma sample dataset from HCC patients and controls. We designed a high-throughput assay using next-generation sequencing and multiplexing techniques, analyzing plasma samples from 554 clinical study participants, including HCC patients, non-HCC cancers, chronic hepatitis B, and healthy controls. HCC detection sensitivity was 84.5% at 95% specificity and 0.94 AUC. Implementing this assay for high-risk individuals could significantly decrease HCC morbidity and mortality.

## Abstract (web summary)

DNA methylation analysis is a promising method to detect liver cancer. Here, the authors develop a 5 CpG site signature which can detect HCC at high specificity across multiple cohorts.

## Introduction

Hepatocellular carcinoma (HCC) is the fifth most common cancer world-wide^1^ and is particularly prevalent in Asia; HCC occurrence is highest in areas where hepatitis B is prevalent which is a high-risk factor for HCC^2^. Follow-up of high-risk populations such as chronic hepatitis patients and early diagnosis of transitions from chronic hepatitis to HCC would improve cure rates. The survival rate of hepatocellular carcinoma is currently quite low because it is almost always diagnosed at the late stages. Liver cancer could be effectively treated with cure rates of >80% if diagnosed early (www.cancer.org/cancer/livercancer). Advances in imaging have improved noninvasive detection of HCC^3,4^. However, current diagnostic methods, which include imaging and immunoassays with single proteins such as alpha-fetoprotein (AFP) often fail to diagnose HCC early because of low accuracy and many early cancers are missed^2^. Moreover, the use of AFP as a tumor marker is limited because of discrepancies among the different methods of measurements^5^) and the most recent guidelines from the American Association for the Study of Liver Diseases (AASLD) discouraged using AFP for surveillance in its update from 2011^6^, however, AFP was shown to detect HCC in subgroups of HCC patients such as patients without HCV infection and in patients with cirrhosis and HIV infection^7^.

These challenges are not limited to HCC but common to other cancers as well. Early detection of cancer in otherwise healthy patients requires noninvasive methods that could be administered to the wide public, are high-throughput and don’t require sophisticated personnel or equipment to administer. Blood is an accessible biological sample and could be drawn in almost any location and shipped to centralized labs for further processing.

Molecular diagnosis of cancer is focused on tumors and biomaterial originating in tumor including tumor DNA in plasma^8,9^, circulating tumor cells^10^ and the tumor-host microenvironment^11–13^. Each of these approaches has its technical limitations. Detection of DNA from tumor origins in cell-free DNA (cfDNA) in plasma is an attractive approach, however, it has its challenges, mainly the small and variable amount of circulating cell-free tumor DNA as well as a requirement for an adequate method to differentiate tumor DNA from cfDNA that originated in other non-malignant tissue in the plasma. Early studies focused on detecting tumor-specific mutations by deep sequencing, however, the variable abundance of specific mutations in tumors resulting from inter- and intra-tumor heterogeneity, which is further limited in the heterogeneous population of cfDNA, reduces the sensitivity of such detection methods^14^. An alternative approach is examining unique tumor DNA methylation profiles by methylation-specific PCR or next-generation sequencing (NGS)^15^. Aberrant DNA methylation profiles are a hallmark of cancer, and a wide body of data has established that tumor DNA methylation profiles are dramatically different than their normal counterparts^16^. Early markers were based on a candidate gene approach utilizing candidate genes hypermethylated in cancer and were based on a limited set of tumor DNA methylation profiles. One of the most successful biomarkers that emerged from this approach is *Sept9* which is used in clinical practice for screening colorectal cancer^17^.

Hypomethylation of HCC DNA is detectable in patients’ blood^18^ and genome-wide bisulfite sequencing was recently applied to detect hypomethylated DNA in plasma from HCC patients^19^. Methylscape is an assay that takes advantage of the global differences in the genomic distribution of methylation positions between cancer and normal tissues, which affect DNA physicochemical properties. The assay utilizes the differential interaction between gold and methylated and unmethylated DNA to detect cancer^20^. Initial studies with the small number of samples suggest that it can detect cancer at close to 0.9 accuracy. However, this tool doesn’t provide information on specific cancer origin, and it is unclear whether the global amount of tumor of cfDNA in plasma will be sufficiently abundant at early stages to be detected by this assay which examines global properties of DNA methylation.

More recently several groups performed a comprehensive comparative analysis of genome-wide DNA methylation profiles of cancer, adjacent tissue DNA and cell-free DNA (cfDNA) in plasma. These studies identified tumor-specific DNA methylation profiles in tumor biopsies and compared them to cfDNA. An immunoprecipitation method with anti-5-methylcytosine antibody which analyzed methylomes of cfDNA in cancer patients revealed thousands of differentially methylated regions in cfDNA^21^. The methylation differences in cfDNA corresponded to DNA methylation differences in the tumors suggesting that DNA methylation signatures in tumor biopsies could be used to identify potential cfDNA tumor markers. A classifier composed of 300 differentially methylated regions (DMR) delineated by machine learning training classified cancer blood samples with high accuracy, sensitivity and specificity and the performance was similar between early and late-stage cancer, suggesting that certain tumor-specific methylation profiles emerge early in cancer and could potentially be used for early cancer detection^21^.

Genome-wide bisulfite sequencing is a relatively costly procedure and requires significant bioinformatics analysis, which makes it unfeasible as a screening tool. The challenge is therefore to delineate a small number of CpGs that could robustly differentiate tumor DNA from a nontumor DNA and to develop a high throughput assay that would enable the screening of wide populations in diverse geographic areas. While this study provides robust proof of principle for the utility of DNA methylation markers in early detection, it still requires 300 DMRs, which makes it difficult to develop into a widely used biomarker in a public health setting. A more recent pan-cancer study has short-listed 100,000 regions as tumor and tissue-specific classifiers and validated them in a large multicenter clinical study. Although the test covers many cancer types it is significantly complex, and its sensitivity was low for early-stage cancers. The study strongly supports the idea that methylation profiles are more sensitive classifiers of cancer in cfDNA than tumor-specific mutations^22^.

In a different approach Xu et al., first compared DNA methylation in hepatocellular carcinoma HCC with blood DNA methylation Illumina arrays using publicly available datasets and established a DNA methylation panel, which was differentially methylated in HCC. This study compared methylation profiles of HCC tumor DNA and normal blood leukocytes and showed that matched plasma cfDNA and tumor DNA are highly correlated. The number of probes was reduced to 10 and they constructed a diagnostic prediction model which had a sensitivity of 85.7% and specificity of 94.3% for HCC in the training data set of 715 HCC and 560 normal samples and a sensitivity of 83.3% and specificity of 90.5% in the validation data set of 383 HCC and 275 normal samples^23^. However, the probes selected were not tested against normal DNA from other tissues that is present in cfDNA or against other cancer types.

The main challenge with many current approaches is that they have not considered cfDNA from other tissues that is found in blood at different levels. Contaminating DNA from another tissue that has a similar methylation profile to a cancer tissue could potentially lead to false positives. In addition, past approaches have quantitatively compared DNA methylation in normal and cancer tissues. This quantitative difference is diluted when tumor DNA is mixed with different and unknown amounts of DNA from other untransformed tissues, which can cause false negatives. These deficiencies in current methods necessitate a different approach.

To address these challenges, we used 12525 methylation profiles in the TCGA (The Cancer Genome Atlas) and GEO data repository collections to delineate DNA methylation positions that are consistently unmethylated in all the samples in the interrogated tissues and blood DNA (Table 1). We then used this shortlist of ubiquitously unmethylated sites to identify sites that were highly methylated in a training set of HCC DNA methylation arrays. These sites exhibit a categorical binary difference between HCC and other tissues, including blood. We shortlisted four CpGs positions that are sufficient to classify HCC in a mixture of normal tissues and blood cells, which we termed “HCC-detect”. We then validated the methylation score composed of these sites in a data set composed of DNA methylation profiles of more than 700 HCC samples. We used a training dataset to discover a single CpG site that was sufficient to differentiate HCC from 31 different cancers and normal cell types, which we termed “HCC-spec”. This was validated on a dataset of 8629 cancer samples from TCGA. The HCC markers were validated on a different data set that used a different method to map DNA methylation in HCC samples and controls and had plasma samples from the patients. The markers were validated also on plasma cell-free DNA. We termed the combination of “HCC-spec” and “HCC-detect” “epiLiver”. The “epiLiver” test classified HCC samples in the public databases with high accuracy. We developed a targeted multiplexed high-throughput next-generation bisulfite sequencing epiLiver test and applied it to plasma samples from 504 individuals from Dhaka city in Bangladesh and another 50 healthy individuals that were available from Innovative^TM^ Research. This test classified HCC patients at 95% specificity and 84.5% sensitivity and detected 75% of early-stage A patients. Our study demonstrates the feasibility of a high-throughput DNA methylation assay that interrogates a small number of CpG sites to classify patients with HCC using a small amount of blood. Translation of such an assay into a routine early detection tool for people who are at high risk for developing HCC could have a significant impact on reducing the morbidity and mortality from HCC in high-risk populations.

**Table 1:** Data sets used for analysis

Data Set	DNA origin	Description	Platform	Public Availability	N
Non-cancer liver samples					***341***
1	Liver	Non-HCC-Diseases	450 K	GSE61258	79
2	Liver	Liver Cirrhosis	450 K	GSE157973	130
3	Liver	Healthy liver tissue	450 K	GSE69852	6
4	Liver	Healthy liver tissue	450 K	GSE76269	10
5	Liver	Adjacent non-tumor tissues	450 K	TCGA	50
6	Liver	Adjacent non-tumor tissues	450 K	GSE54503	66
Non-cancer non-liver samples					***909***
7	Various tissues	Adjacent non-tumor tissues	450 K	TCGA	677
8	Lung	Normal	450 K	GSE63704	26
9	Ovaries	Normal	450 K	GSE65821	6
10	Stomach	Normal	450 K	GSE85464	19
11	Airway epithelial cells	Normal	450 K	GSE85566	115
12	Breast tissue	Mamoplastic reduction	450 K	GSE60185	15
13	Pancreas	Normal	450 K	GSE53051	12
14	Kidney, prostate, bladder	Normal	450 K	GSE52955	16
15	Colon	Normal	450 K	GSE42752	19
16	17 somatic tissues	Normal	450 K	GSE50192	4
BLOOD					***968***
17	Blood	Healthy	450 K	GSE40279	656
18	Blood	Healthy	450 K	GSE61496	312
Liver Cancer samples					***739***
19	Liver	Liver Cancer	450 K	TCGA	380
20	Liver	Liver Cancer	450 K	GSE76269	227
21	Liver	Liver Cancer	450 K	GSE75041	66
22	Liver	Liver Cancer	450 K	GSE54503	66
Non-liver Cancer samples					***8961***
23	Nonliver cancer tissues	31 types of non-HCC Cancers	450 K	TCGA	8961
Plasma samples					**737**
24	Plasma	cfDNA	MCTA-Seq	GSE63775	183
25	Plasma	cfDNA	MiSeq	This study	554

## Results

### Delineating ubiquitously methylation-resistant CpG sites in blood and normal tissues

We identified CpG positions in publicly available DNA methylation arrays that are uniformly unmethylated across all the individuals and across 17 different somatic tissues.

These selected “methylation resistant” sites were highly enriched for CpG islands (10xe^−814^, Hypergeometric test), Transcription Start sites TSS200(7.7xe^−317^), 1^st^ exon (3xe^−68^), 5’UTR(3.8xe^−27^), and Phantom High CpG density promoters (5.22 fold enrichment 5.6xe^−395^) but depleted for the north and south shores of CpG islands (3xe^−28^ and 3xe^−20^), enhancers (a 4.47 fold depletion 6.8xe^−145^), 3’UTR (a 13 fold depletion 3.8xe^−36^) and low CpG density Phantom promoters (a 2.67 fold depletion 6.3xe^−5^).

### Discovery and validation of “HCC-detect”

**Fig. 1:**
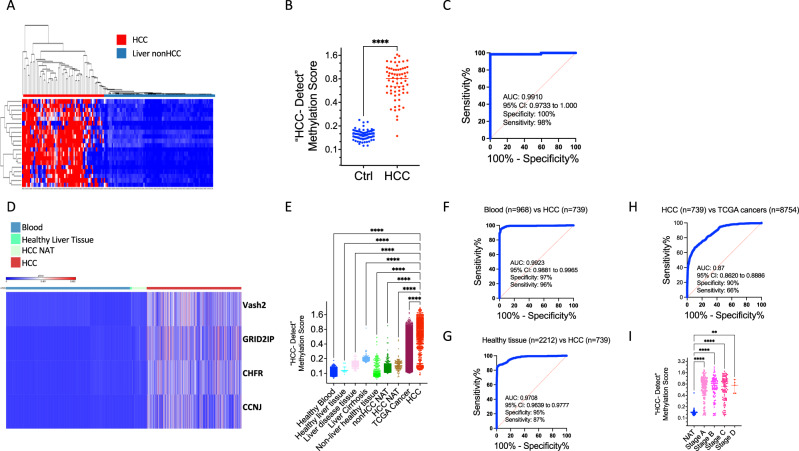
Training and validation of “HCC-detect” DNA methylation marker set. **A** Heatmap and hierarchical clustering showing methylation levels of top 20 CpGs that are categorically different between noncancer liver samples (fibrosis) (*n* = 79) and HCC samples (*n* = 66) in the training cohort (GSE61258, GSE54503). **B** Scatterplot of “HCC-detect” methylation scores calculated for HCC samples (*n* = 66) and controls (*n* = 79) in the training cohort (*p* < 0.0001, Man-Whitney test, two tailed), The line at the median with 95% confidence interval is also shown in the plot. **C** ROC curve of “HCC-detect” methylation scores classifying blood and HCC samples from the training cohort. **D** Heatmap of methylation values for the four CpG sites included in “HCC-detect” in the validation cohort of blood (*n* = 968), heathy liver (*n* = 16), liver NAT (*n* = 116) and HCC samples (*n* = 739) from TCGA and GEO data repository (see Table 1 for details). **E** Scatterplot of “HCC-detect” Methylation score (each spot represents one sample) in healthy blood (*n* = 968), healthy liver tissue (*n* = 16), liver disease (*n* = 79), liver cirrhosis (*n* = 130), non-liver healthy tissues (*n* = 234), NAT to non-HCC tumors (*n* = 723), NAT to HCC (*n* = 116), 31 cancers in TCGA (*n* = 8754) and HCC (*n* = 739). The statistical analysis was performed using Kruskal-Wallis nonparametric ANOVA (two-sided) with Dunn’s multiple comparisons to compare each group to the HCC group. The statistical analysis revealed a significant difference between the HCC group and all other groups (F = 793, DF 11696; *p* < 0.0001 for all comparisons). The line at the median with 95% confidence interval is shown in the plot **F** ROC curve of “HCC-detect” methylation scores classifying healthy blood (968) and HCC samples (739) in the validation cohort. **G**. ROC curve of “HCC-detect” methylation scores classifying healthy tissues (2212) and HCC samples (739) in the validation cohort. **H** ROC curve of “HCC-detect” methylation scores classifying HCC samples (739) and 8754 samples from 31 different types of cancer (Supplementary Table 1). **I** The median +/− methylation scores for “HCC-detect” with 95% confidence intervals in HCC NATs (*n* = 50) and different stages of HCC in the validation cohort from TCGA (Stage A = 175, Stage B = 87, Stage C = 86, Stage D = 5). The statistical analysis was performed using Kruskal-Wallis nonparametric ANOVA (two-sided) with Dunn’s multiple comparisons test, with statistical significance indicated as **p < 0.001 and ****p < 0.0001. The line at the median with 95% confidence interval is shown in the plot. Source data are provided as a Source Data file.

Remarkably, many of these ubiquitously methylation-resistant CpGs were methylated in the HCC discovery cohort (*n* = 66) and not in non-HCC liver samples, which include cirrhosis (*n* = 79). 286 CpG positions were methylated more than 20% in at least 50% of the HCC samples but not in the non-HCC samples. A list of the top 20 CpG sites (heatmap Fig. 1A) was further reduced by penalized regression to four CpG sites; cg02012576 an intergenic region associated with the Checkpoint With Forkhead And Ring Finger Domain (CHFR) gene, cg03768777 at the 1^st^ exon of the Vasohibin 2 (*VASH2*) gene, cg05739190 at the 1^st^ exon of the Cyclin-J gene (*CCNJ*) and cg24804544 at the body of the Glutamate Receptor, Ionotropic, Delta 2 (*GRID2IP*) Interacting Protein 1 gene (*GRID2IP*). A weighted polygenic methylation score significantly differentiated HCC and non-HCC control samples (Fig. 1B). A Receiver Operating Characteristic Curve (ROC) analysis performed on the calculated polygenic methylation scores for the HCC and control samples shows an area under the curve of 0.9910 (Fig. 1C). Our training cohort included HCC samples from all stages with the goal of broad detection of cancer notwithstanding stage. We termed the four CpG marker set, “HCC-detect”.

“HCC-detect” was validated using DNA methylation 450 K array data for 739 HCC, 116 adjacent non-tumor tissues (NATs), 16 healthy liver and 968 blood samples (heatmap Fig. 1D). “HCC-detect” score (as described in the methods) was computed for 11718 samples in the public data and it significantly differentiates HCC from all other groups (Fig. 1E) (Table 1 and Supplementary Table 1). (Fig. 1E). ROC analyses computed for different comparisons show excellent biomarker properties; AUC of 0.99 for HCC versus Healthy blood (Fig. 1F), AUC of 0.97 for HCC versus all healthy and NAT tissues including liver (Fig. 1G), AUC of 0.95 for HCC samples are compared to healthy tissues (specificity-95%; sensitivity-85%), AUC of 0.92 for HCC compared to NAT (specificity-94%; sensitivity-95%), AUC of 0.966 for HCC versus healthy liver tissue (specificity-100%; sensitivity- 88%) and AUC of 0.87 for HCC versus 31 different types of cancer (Supplementary Table 1) (Fig. 1H). The “HCC-detect” methylation score detects early-stage HCC samples as well as late-stage HCC (Fig. 1I). Similar AUC values were obtained when equal weight was given to each CpG in the detect score assuming that methylation at any of the four CpGs is sufficient to classify a sample as HCC, though certain CpGs are methylated (>20%) in a higher fraction of HCC samples than others (59% for *VASH2*, 57% for CHFR, 50% for *GRID2IP* and 44% for *CCNJ*) (Supplementary Table 2).

### Discovery and validation of “HCC-spec”

**Fig. 2:**
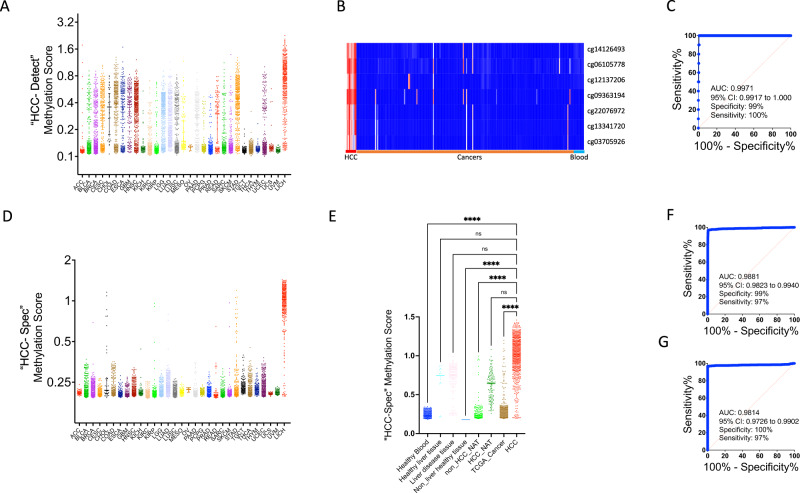
Training and validation of “HCC-spec” DNA methylation marker set. **A** Scatterplot of “HCC-detect” methylation scores in 31 different cancer types and HCC (Supplementary Table 1 for acronyms and number of samples). The line at the median with 95% confidence interval is also shown in the plot. **B** Heatmap of methylation levels of top 7 CpGs shortlisted for discriminating HCC (*n* = 10) from other cancers (10 randomized samples from each of 16 different cancers, Esophageal carcinoma, Head and Neck squamous cell carcinoma, Testicular Germ Cell Tumors, Bladder Urothelial Carcinoma, Brain Lower Grade Glioma and Glioblastoma multiforme, Breast invasive carcinoma, Colon adenocarcinoma, Prostate adenocarcinoma, Stomach adenocarcinoma, Lung adenocarcinoma, Cervical squamous cell carcinoma and endocervical adenocarcinoma, Pancreatic adenocarcinoma, Kidney renal clear cell carcinoma, Kidney renal papillary cell carcinoma, Kidney Chromophobe, Skin Cutaneous Melanoma, and 10 blood) in the training cohort. **C** ROC curve of “HCC-spec” methylation scores HCC (*n* = 10) and other 16 kinds of cancer samples in the training cohort (*n* = 210). **D** Scatterplot of “HCC-Spec” methylation scores in 31 different cancer types and HCC (Supplementary Table 1 for acronyms and number of samples per cancer). The line at the median with 95% confidence interval is also shown in the plot**. E** Scatterplot of “HCC-spec” Methylation scores (each spot represents one sample) in healthy blood (n = 968), healthy liver tissue (*n* = 16), liver disease (*n* = 79), non-liver healthy tissues (*n* = 234), NAT to non-HCC tumors (*n* = 723), NAT to HCC (n = 116), 31 cancers in TCGA (*n* = 8754) and HCC (*n* = 739) (****p < 0.0001, n.s. nonsignificant, Kruskal-Wallis nonparametric ANOVA (two-sided) with Dunn’s multiple comparisons test). The line at the median with 95% confidence interval is also shown in the plot. **F** ROC curve of “HCC-spec” Methylation score classifying 31 other cancers (*n* = 8754) and HCC samples (739) in the validation cohort. **G**. ROC curve of “HCC-spec” Methylation score classifying all blood samples (*n* = 968) and HCC (*n* = 739) samples in the validation cohort. Source data are provided as a Source Data file.

“HCC-detect” score developed for HCC preferentially detects HCC amongst 31 cancers in TCGA (Fig. 2A), however, it detects other cancers as well, reducing the specificity and sensitivity of differentiating HCC from other tumors (specificity 90% and sensitivity 66%) (Fig. 1H). Using 230 randomly selected DNA methylation samples from TCGA representing 17 different cancers (210 samples), 10 HCC samples and 10 healthy blood samples we shortlisted 7 CpGs that differentiate HCC and tumors from other origins (Heatmap in Fig. 2B); cg14126493 at the body of the *F12* gene had the largest effect which was designated “HCC-spec”. A weighted methylation score for *F12* computed by a linear regression equation (Supplementary Table 3) classified all HCC samples correctly within a mixture of 240 samples in the training cohort. ROC curve (HCC versus all other samples) revealed an AUC of 0.9973 (sensitivity-99%; specificity-100% (Fig. 2C).

“HCC-spec” was validated by classifying HCC DNA within a mixture that included 31 different tumors, nonmalignant tissues, and HCC samples (*n* = 11,918) (Supplementary Table 1) (scatterplot in Fig. 2D). The “HCC-spec” score is significantly different between HCC and healthy blood, healthy tissues, adjacent non-tumor tissues (NATs) and other cancers but is not significant between HCC and healthy liver tissue and liver-disease samples (Fig. 2E). AUC for HCC versus all other cancers is 0.988 (Fig. 2F), versus normal blood is 0.981 (Fig. 2G) and versus healthy tissues is 1. AUC for HCC versus non-malignant liver DNA is much lower; for healthy liver tissue it is 86% (specificity-100%; sensitivity-73%) and for liver disease tissues it is 0.84 (specificity-95%; sensitivity-71%).

The AUC for a combined score (HCC-spec + HCC-detect) for HCC against all other samples (tumor and normal) is 0.9862 (Fig. 2G). At the threshold calculated by this ROC (a combined score of 0.87) other liver tissues and liver disease DNA will be detected at the rate of 50%. A higher threshold of a combined score of 1.1 differentiates HCC from other liver diseases (AUC is 0.937; sensitivity-87%; specificity-95%).

“HCC-detect” classifies cholangiocarcinoma cases as cancer, AUC of ICC (*n* = 39) against healthy blood (*n* = 968) is 0.9875 (sensitivity-97%; specificity-95%) (Supplementary Fig. 1A), however, “HCC-spec” does not differentiate ICC and blood with AUC 0.5436 (sensitivity-17%; specificity-95%) (Supplementary Fig. 1B) and is less sensitive in classifying “ICC from NAT, AUC of 0.833 (sensitivity-67%; and specificity-100%) (Supplementary Fig. 1A).

There are no significant differences in “HCC-detect” and “HCC-spec” methylation scores in HCC TCGA dataset between white, Asians, and Black or African Americans (the ethnicity terms are derived from the TCGA terminology and definitions). (Supplementary Fig. 2).

**Fig. 3:**
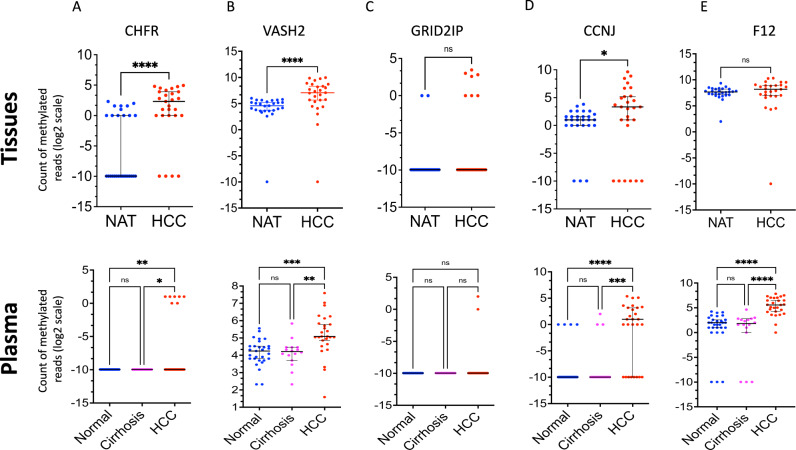
Validation of differential methylation of “HCC-spec” and “HCC-detect” CpG sites in the GSE63775 dataset. The top panel shows the count of methylated reads in genomic regions containing each of the 5 CpGs of the markers for tumor tissue samplesfrom HCC (*n* = 27), NAT (*n* = 27), while the bottom panel shows the same for plasma samples from HCC (*n* = 27), cirrhosis (*n* = 16), and NAT (*n* = 29). The CpGs analyzed were from **A**. *CHFR*, **B**. *VASH2*, **C**. *GRID2IP*, **D**. *CCNJ*, and **E**. *F12*. To evaluate significance in tumor tissues, we used a nonparametric two-tailed t-test. For plasma samples, we used Kruskal-Wallis nonparametric ANOVA with Dunn’s multiple comparisons test (****p < 0.0001, ***p < 0.001, **p < 0.01, *p < 0.05, n.s. nonsignificant). The data points were plotted on a log2 scale on the y-axis, and each zero value on the y-axis was replaced with 0.001 to avoid undefined logarithm. The line at the median with 95% confidence interval is shown in all plots. Source data are provided as a Source Data file.

The genes included in “HCC-detect” and “HCC-spec” were significantly differentially methylated in a different validation set that had HCC cell free plasma DNA and tumor samples compared to cirrhosis and normal liver (GSE63775^24^) (Fig. 3A, B, D, E) except for *GRID2IP* which was significantly differentially methylated in HCC tissue samples (Fig. 3C) but didn’t reach significance in plasma because of the low number of reads in the serum sample (Fig. 3C). Importantly cirrhosis samples in either tumor or plasma were unmethylated as well.

**Fig. 4:**
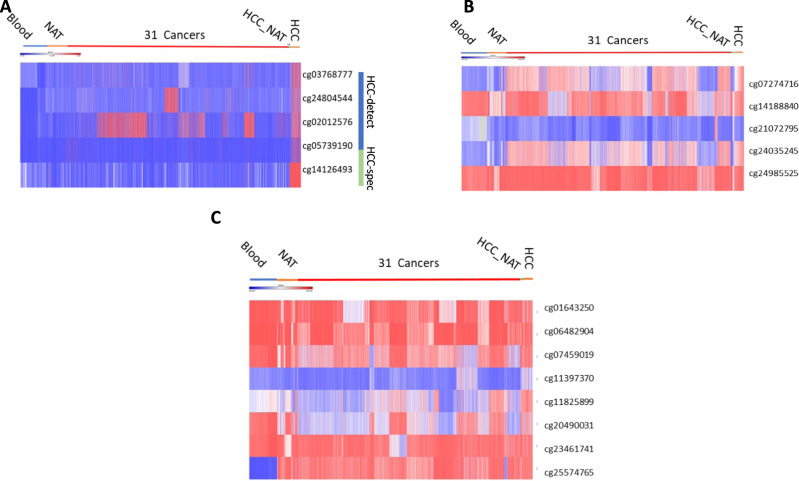
Comparisons of cancer type specificity of “HCC-detect and spec” combination and two previously published biomarkers. Heatmap presentation of the methylation values for shortlisted HCC detection CpG sites described here (**A**) in Liu et al. (*22*) (**B)** and Zhang et al., **(C)** (*19*). Source data are provided as a Source Data file.

We compared “HCC-spec” and “HCC-detect” DNA methylation markers (Fig. 4A) to two other highly promising sets of HCC biomarkers that were recently described^22,25^ (Fig. 4B, C) using DNA methylation values for the respective CpGs in Illumina 450 K arrays from the 11701 samples described above. The heatmaps presented in Fig. 4 show that although previously published markers display dramatic differences in methylation between HCC and HCC-NAT samples, there is a high background of DNA methylation across other cancers and normal tissues. The combined “HCC-detect” and “HCC-spec” markers delineated here show a categorical differentiation between high methylation in HCC and notably low methylation in other tissues and most cancers. While two of the “HCC-detect” markers are methylated to different extent in several cancers (Fig. 4A, B), *F12* “HCC-spec” is exquisitely methylated in HCC and liver disease samples but not in other cancers, normal tissues or blood (Fig. 4D).

### Validation of “HCC-detect” and “HCC-spec” DNA methylation in a clinical study of plasma cfDNA (ClinicalTrials.gov ID: NCT03483922; *n* = 554)

**Fig. 5:**
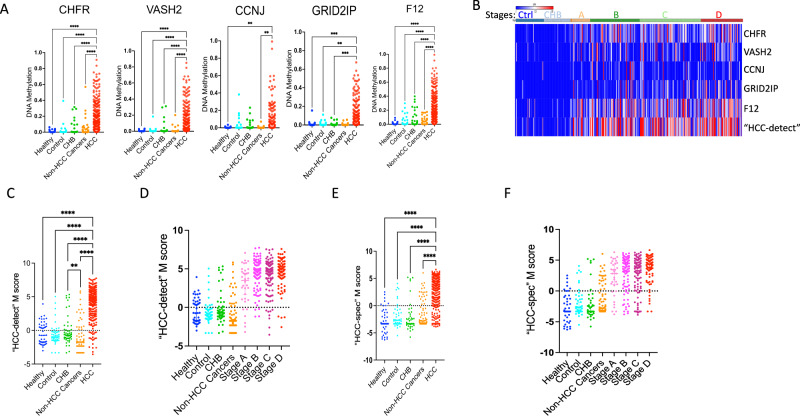
Validation of “HCC-detect” and “HCC-spec” in plasma samples in the Dhaka clinical study and healthy plasma from Innovative^TM^ Research. **A** Differential methylation of CpGs included in the “HCC-detect” markers (cg02012576 (*CHFR*), cg03768777 (*VASH2*), cg05739190 (*CCNJ*), cg24804544 (*GRID2IP*) and “HCC-spec” cg14126493 (*F12*) in healthy controls (*n* = 46), CHB (*n* = 49), HCC (302). Beta values for each sample are presented in the scatterplot. **B** Heatmap depicting the median methylation for each of the 5 regions amplified included in “HCC-detect” and “HCC-spec”. “HCC-detect” row shows the sum of median methylation values for each sample. The color codes for the groups are listed in the legend. **C** Scatterplots of the “HCC-detect” M scores for control (*n* = 46), healthy plasma (*n* = 50), CHB (*n* = 49), non-HCC cancers (*n* = 102) and HCC (302). **D** Median “HCC-detect” M scores for healthy controls (*n* *=* 46), healthy plasma (*n* = 50), CHB (*n* = 49), non-HCC cancers (*n* = 102) and four stages of HCC (Stage A + O- 34, Stage B-86, Stage C-106 and Stage D-76) + /− 95% confidence interval. The Supplementary Table 4 shows the statistical significance comparisons between the groups. The line at the median with 95% confidence interval is shown in the plot**. E** Scatterplots of the “HCC-spec” M scores for healthy plasma, control, CHB, non-HCC cancers and HCC. **F** Median “HCC-spec” M scores for healthy plasma, control, CHB, non-HCC cancers and four stages of HCC + /− 95% confidence interval. The Supplementary Table 5 shows the statistical significance for comparisons between the groups. The line at the median with 95% confidence interval is shown in the plot. Significance in all tests was determined by Kruskal-Wallis nonparametric one-way ANOVA with Dunn’s multiple comparisons test (****p < 0.0001, ***p < 0.001, **p < 0.01, *p < 0.05). Figures A, C and E only show scatterplots for comparisons with a *P* value of 0.05 or less, indicating that differences between groups are only considered significant if they meet this threshold. Any comparisons with a higher *P* value are not displayed. Source data are provided as a Source Data file.

**Fig. 6:**
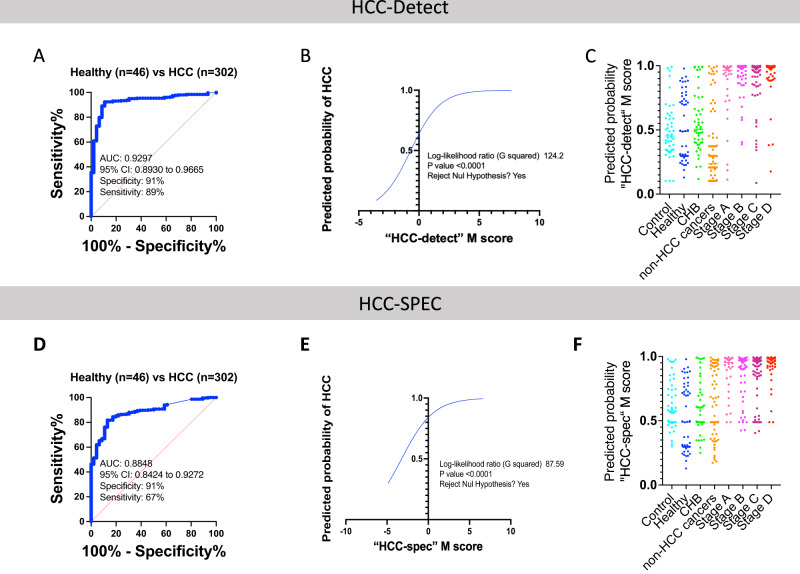
Biomarker characteristics of “HCC-detect” and “HCC-spec” M scores. **A** ROC curve of “HCC-detect” M scores classifying HCC in healthy control (*n* = 46) and HCC cases (*n* = 302) in the Dhaka clinical study. **B** Logistic regression curve plotting the predicted probability of HCC as a function of “HCC-detect” M score. **C** Predicted probabilities (0 to 1) for each of the samples from the Dhaka clinical study and healthy plasma from Innovative^TM^ Research calculated using the logistic regression equation for the “HCC-detect” M scores. The Supplementary Table 6 shows the statistical significance for comparisons between the groups. **D** ROC curve of “HCC-spec” M score classifying healthy control (*n* = 46) and HCC cases (*n* = 302) samples in the Dhaka clinical study. **E** Logistic regression curve plotting the predicted probability of HCC as a function of “HCC-spec” M score. **F** Predicted probabilities (0 to 1) for each of the samples from the Dhaka clinical study healthy plasma from Innovative^TM^ Research calculated using the logistic regression equation for the “HCC-spec” M score. The Supplementary Table 7 shows the statistical significance for comparisons between the groups. The clinical study included (controls (*n* = 46), CHB (*n* = 49) and four stages of HCC (Stage A + O- 34, Stage B-86, Stage C-106 and Stage D-76). Significance was determined by Kruskal-Wallis nonparametric one-way ANOVA with Dunn’s multiple comparisons test (****p < 0.0001, ***p < 0.001, **p < 0.01, *p < 0.05, n.s. nonsignificant). Source data are provided as a Source Data file.

The CpG positions within the genes we selected demonstrated differential methylation patterns in plasma cfDNA from HCC patients in comparison to plasma from healthy people or from people with chronic hepatitis B, except for *CCNJ*, which showed significantly higher methylation levels in HCC patients compared to healthy people and non-HCC cancer patients. In controls and chronic hepatitis B patients, median methylation was slightly above 0%, while HCC samples showed methylation levels ranging from 50% to 80%. (Fig. 5A scatterplots). Hypermethylation was observed even at early HCC stages (Supplementary Fig. 3A), similar to the results obtained in the TCGA HCC dataset (Fig. 1I). We noted a high correspondence in methylation levels of the CpG included in the “HCC-detect” and “HCC-spec” sets and proximal CpGs included in the targeted sequence (heatmap Supplementary Fig. 3C). The median methylation in the amplified region was compared for each of the 5 genes (heatmap Fig. 5B). “HCC-detect”, and “HCC-spec” M scores (calculated as described in the methods) significantly differentiated the HCC group from either the healthy control or chronic hepatitis B groups (Fig. 5C, E) but there was no significant difference between the chronic hepatitis B (CHB) and both healthy control groups. Importantly, both the “HCC-detect” M scores and the “HCC-spec” M scores were significantly different from control and chronic hepatitis B groups at early and late HCC stages (Fig. 5D, F, Supplementary Tables 4 and 5) but there is no significant difference between HCC stages. The AUC for the “HCC-detect” score (302 HCC patients and 46 controls) was 0.93, the specificity 91% and sensitivity 89% (Fig. 6A). The 95% bootstrapped (nonparametric) confidence interval for “HCC-detect” and for “HCC-spec” were calculated for healthy controls and Stage A, Stage B, Stage C and Stage D using 1000 bootstrap replicates. The confidence intervals and corresponding *p* values were (−4.73, −2.87), *p*-value = 9.12e-251 and (−5.57, −3.41), *p* value = 1.49e-253 for “HCC-detect” and “HCC-spec” for healthy controls and Stage A; (−5.72, −4.72), *p* value ~ 0 and (−5.98, −4.52), *p* value = 4.6e-315 for “HCC-detect” and “HCC-spec” for healthy controls and Stage B; (−2.55, 2.73), *p* value ~ 0 and (−5.85, −4.53), *p* value ~ 0 for “HCC-detect” and “HCC-spec” for healthy controls and Stage C; (−5.99, −4.96), *p* value ~ 0 and (−6.82, −5.6), *p* value ~ 0 for “HCC-detect” and “HCC-spec” for healthy controls and Stage D. The *p* values indicate the levels of significance for rejecting the null hypothesis of no correlation. Cross-validation (as described in the methods) using models obtained from three randomly sampled training dataset reveals that the “HCC-detect” and “HCC-spec” predictions were significantly different between the HCC group and either the healthy control or chronic hepatitis B (Supplementary Fig. 4, Supplementary Table 11).

Logistic regression modelled “HCC-detect” M score as a predictor of probability of HCC (Fig. 6B) and a predicted probability for each person was computed using the logistic regression equation (scatterplot Fig. 6C, Supplementary Table 6). The HCC samples cluster around the probability of 1, few CHB samples are predicted a probability of 1 while most of the samples of the healthy and CHB samples median is around a predicted probability of 0.5. The AUC for the “HCC-spec” M score is 0.89 with specificity of 91% and sensitivity of 67% (Fig. 6D). We computed the logistic regression equation for “HCC-spec” M- scores (Fig. 6E) and the predicted probability for each person (Fig. 6F, Supplementary Table 7).

**Fig. 7:**
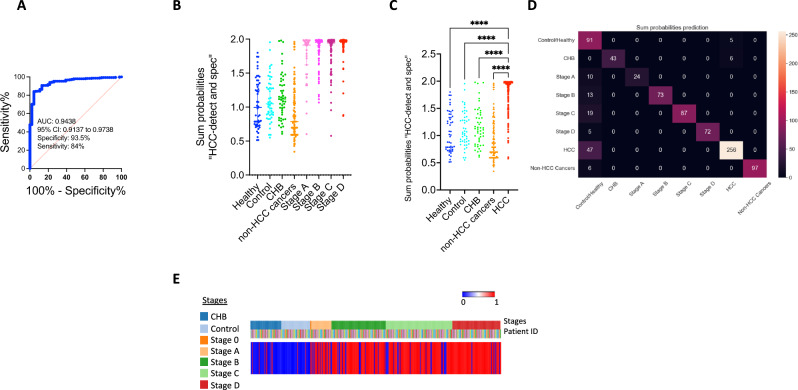
Classification of HCC by a combined “HCC-detect” and “HCC-spec” probability score (“epiLiver”). **A** ROC curve of “HCC-detect and spec” sum probabilities score as a classifier of HCC (healthy plasma (n = 50), healthy controls, *n* = 46, CHB (*n* = 49), non-HCC cancers (*n* = 102) and HCC cases, *n* = 302) in the Dhaka clinical study. **B** Median +/− 95% confidence interval for the sum probabilities scores for the 50 healthy plasma (Innovative^TM^ Research), healthy controls, chronic hepatitis B 102 non-HCC cancer patients and four stages of HCC. Kruskal-Wallis nonparametric one-way ANOVA with Dunn’s multiple comparisons test was used to compare the groups. Statistical significance comparisons between the groups are shown in Supplementary Table 8. **C** Scatterplot of the sum probabilities scores for each of the samples in the control, CHB and HCC groups. Figure show scatterplot for comparisons with a *P* value of 0.05 or less, indicating that differences between groups are only considered significant if they meet this threshold. Kruskal-Wallis nonparametric one-way ANOVA with Dunn’s multiple comparisons test was used to compare the groups, with statistical significance indicated as ****p < 0.0001. Any comparisons with a higher *P* value are not displayed. **D** Confusion matrix representing the classification (1,0) of HCC samples, based on their inclusion in the control, CHB, and HCC stages groups (for confusion matrix visualization, we used “seaborn” package” (10.5281/zenodo.7823382). **E** Heatmap presentation of HCC classifications for each of the samples in the Dhaka study. Samples were classified either as HCC (red) or nonHCC (blue). Stages are color coded as indicated in the legends. Patient IDs are color coded as indicated. In the scatterplots, only comparisons with *P* value less than or equal to 0.05 are displayed, otherwise difference between the groups is not significant. Source data are provided as a Source Data file.

We computed ROC curve of the combined probability of “HCC-detect” and “HCC-spec” scores of 46 healthy controls and 302 HCC patients which we termed “epiLiver” (Fig. 7A). The calculated AUC is 0.94 the specificity is 95% and the sensitivity is 84%. The median score for each of the HCC stages including early stages are not different from each other (Fig. 7B, Supplementary Table 8 and a scatterplot for all individual samples in Fig. 7C). We calculated a threshold sum probability from the AUC curve (1.62) and used it to classify the samples as either HCC (1) or no HCC (0) (scatterplot Fig. 7D). This threshold accurately classifies 93.5% of the control samples 71% of the Stage A samples, 86% of the Stage B samples, 82% of the Stage C samples and 92% of the Stage D samples (heatmap presenting the classification for each of the 397 samples (HCC-red, no HCC-blue) is presented in Fig. 7E). Using this threshold, 12% of the chronic hepatitis B (CHB) samples are classified as HCC compared with 5% of controls.

### Comparing epiLiver markers, AFP and their combination as predictors HCC

**Fig. 8:**
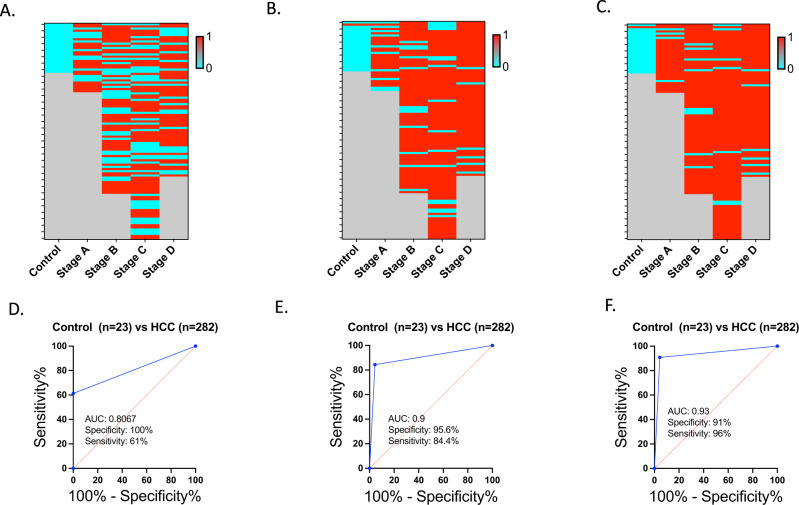
HCC prediction with AFP and epiLiver. Heatmap (**A**) and ROC curve (**D**) show the prediction of HCC using AFP with a threshold of 400 ng/mL. Heatmap (**B**) and ROC curve (**E**) display the prediction of HCC through the sum of probabilities (threshold = 1.62) for samples with AFP data. Heatmap (**C**) and ROC curve (**F**) illustrate the combined prediction of HCC by using both AFP with a threshold of 400 ng/mL and the sum of probabilities (threshold = 1.62). The heatmaps use the color cyan to represent the absence of prediction, while the color red represents prediction. Grey is used to indicate no values.Source data are provided as a Source Data file.

100% of the control samples were classified as negative by AFP and 61% of HCC were classified properly (63% Stage A, 59% Stage B, 58% Stage C, 68% Stage D) (Fig. 8A prediction, Fig. 8D ROC). The combined “sum of probabilities” score for EpiLiver classified 96% of the control samples as negative and 85% of HCC as positive (72% of Stage A, 85% Stage B, 84% of Stage C, and 92% Stage D) (Fig. 8B prediction, Fig. 8E ROC). A combined epiLiver and AFP prediction classifies 96% of the control samples as negative and, 88% of HCC samples as positive (88% of Stage A, 89% of Stage B, 93% of Stage C, and 92% of Stage D).

## Discussion

The main challenge for cfDNA-based cancer prediction is the limited amount of tumor DNA in plasma and its dilution with DNA from blood and other tissues. Although recent technological advances cited above have boosted the feasibility of this approach, cfDNA still possess a formidable challenge particularly its translation into a widespread health management instrument which needs to be economical, high-throughput and specific for cancer type to have an impact on public health. DNA methylation profiles can accurately differentiate tumor DNA from adjacent non-tumor tissues and blood. However, tumor DNA methylation profiles might be like the pattern of methylation in unrelated tissues. Since the origin of DNA in cfDNA is unpredictable and its relative abundance is unknown, this could potentially confound the results and lead to false positives. In addition, quantitative differences in DNA methylation which are clearly sufficient to differentiate between relatively homogenous samples are confounded in plasma where mixtures of DNA from different origins with different levels of methylation coexist. Although algorithms for deriving cell of origin from DNA methylation profiles were developed, they require a relatively large number of sites which is unfeasible for a high-throughput and inexpensive public health tool. An additional complicating factor is that several cancer DNA methylation biomarkers developed to date could detect several tumors beyond HCC (Fig. 4B, C), thus providing no direction as to the treatment response to the potential cancer detection. In order to address these challenges, we set a path to discovery that was based on the following guidelines: First, reduce cost and increase high-throughput by discovering a small number of robust DNA methylation positions which are amenable to simple targeted amplification and multiplexed NGS. By multiplexing a few hundred samples in one run the sequencing costs are reduced to single USD per assay. Moreover, sequencing of the entire region provides a state of methylation of several sites in a region increase the accuracy of detection by ruling out spurious methylation events of single sites which is not possible with single site-specific methylation-specific PCR assays. Second, reduce background of normal tissue DNA by focusing on sites that are ubiquitously methylation resistant across tissues and blood. Third, combine markers that detect cancer with markers that identify the tissue of origin. Markers of tissue of origin are different than cancer-specific markers and do not necessarily differentiate cancer from normal cells from the same tissue of origin. Hence, the need for using two different strategies for discovery of markers that detect cancer and others that identify the tissue of origin.

We demonstrate here a shortlist of methylation-resistant sites that are ubiquitously unmethylated across hundreds of individual samples in 17 tissues and blood. These sites are highly enriched in CpG-dense promoters and depleted in enhancers and other genomic features. The depletion of methylation in certain genomic features has been recognized since the discovery of CpG islands four decades ago^26^ and was further confirmed by genome-wide bisulfite mapping studies^27^. We show here that some of these highly DNA methylation-resistant CpGs are consistent across hundreds of people and thus offer a clean background. Using a training cohort from GSE54503 DNA methylation collection, we show that a fraction of these highly methylation-resistant sites is methylated in HCC tumors (Fig. 1A). With main goal of cost efficiency and high-throughput potential we determined the smallest number of CpGs that are sufficient to serve as a classifier of HCC with great accuracy. We show that methylation of only four CpG positions is sufficient to call 98% of the HCC samples with 100% specificity. Although not all HCC samples have all 4 positions methylated, 98% of the samples have at least one CpG methylated. We proposed that these four CpGs serve as “HCC-detect” set of markers. We validated that these markers could classify HCC accurately with high sensitivity and specificity using a dataset of 739 HCC and samples from 2212 healthy tissues. Importantly, these markers can differentiate HCC from other liver diseases such as cirrhosis (Figs. 1E and 3) and chronic hepatitis B (Figs. 5 and 7). However, as expected when “HCC-detect” was used against a panel of 31 cancers from other tissue origins these cancers were detected at different sensitivities (Fig. 2A) and it detects cholangiocarcinoma (Supplementary Fig. 1) a bile duct cancer that is found in the liver but is of different histopathological origin. To provide a classifier of liver origin tumor we discovered a single DNA methylation biomarker that distinguished DNA from the liver origin from other tissues, including cholangiocarcinoma (Supplementary Fig. 1), which we termed “HCC-spec”. A combined test of “HCC-spec” and “HCC-detect” provides a highly accurate classification of HCC and distinguishing it from other tumors providing clear guidance on the location of the tumor, which will speed up applying the necessary follow-up and clinical measures. “HCC-spec” differentiates Importantly, both “HCC-detect”, and “HCC-spec” markers are methylated at the early stages of HCC and remain methylated to the most advanced stages. This will provide a broad-spectrum single test for first screening of asymptomatic patients notwithstanding the stage of cancer avoiding missing patients who have moved to a later stage or who are at an earlier stage of the disease.

“HCC-detect” and “HCC-spec” markers were discovered and validated in tumor biopsy material, it is important to determine whether they are methylated in plasma cfDNA as well. We therefore validated the methylation state of these markers in a previously published data set GSE63775 that examined genome-wide methylation of HCC in tumor and plasma in a completely different cohort and used a different platform for DNA methylation mapping (bisulfite conversion followed by methylated CpG tandem amplification and sequencing) (Fig. 3A-E)^24^. These data are consistent with other studies that showed correspondence between DNA methylation profiles of tumor tissue and plasma cfDNA^22,23^.

We applied the agnostic criteria of ubiquitous methylation resistance in normal tissues and methylation in HCC for selecting the “HCC-detect” biomarker set, irrespective of biological function. Nevertheless, the fact that we found a set of CpGs that are methylated in a large subset of HCC patients and at all stages of HCC suggests that these DNA methylation events might be important for the early stages of the disease as well as its maintenance. The fact that no single marker is methylated in all normal samples but at least one of the 4 markers is methylated in almost all HCC patients suggests that at least one of the 4 markers is essential. Indeed, the “HCC-detect” CpGs are associated with genes involved in cell cycle regulatory events, EMT transition and angiogenesis. CHFR encodes a cell cycle (G2/M) checkpoint, which was suggested to be a tumor suppressor gene^28^; methylation of CHFR was associated with noninvasive colorectal cancer^29^, esophageal carcinoma^30^, hepatocellular carcinoma^31^, higher grade gastric cancer^32^ and non-small cell lung carcinoma^33^. Vasohibin 2 *(VASH2*) gene was implicated in angiogenesis in invasive tumors^34^ and was previously shown to be methylated in HCC^35^. *VASH2* is a promoter of an angiogenesis pathway it is therefore expected to promote cancer and indeed higher expression of *VASH2* is associated with poor progression of pancreatic cancer^36^, esophageal squamous cell carcinoma^37^, breast cancer^38^ and epithelial-mesenchymal transition (EMT) in HCC^39^. We would, therefore, expect hypomethylation of the 5’regulatory region of the gene rather than the observed hypermethylation of the 1^st^ exon. We don’t know how this methylation event is correlated with expression and it needs to be further explored. Cyclin-J gene (*CCNJ*) encodes the cyclin J protein, which was proposed to be involved in early embryonic division cycles of Drosophila^40^ and was previously found to be methylated in liver cancer^41^. Glutamate Receptor, Ionotropic, Delta 2 (GRID2) Interacting Protein 1 gene (*GRID2IP*) is expressed in the brain it interacts with Glutamate receptor delta-2 and was shown to be involved in synaptogenesis in fiber-Purkinje cells^41^. However, its role in cancer has not yet been fully explored. The methylated CpG included in our detect marker set in a CpG island in the body of the gene and it is unclear whether this CpG island has any regulatory role. The role of this gene in HCC remains to be determined. The *F12* gene encodes coagulation factor XII^42^. The CpG position in a CpG-rich island in the body of this gene and was selected here only for its exquisite HCC-specific methylation and methylation resistance in 31 other tumors (Fig. 2D). The relationship between methylation and expression is unclear and needs to be further explored.

We then examined whether these data could be applied as a high-throughput assay to detect HCC in a clinical setting by examining plasma DNA from 402 people, which included all stages of HCC as well as healthy controls and chronic hepatitis B patients that are at high risk of conversion to HCC. By targeting a small number of amplicons, we significantly reduce the cost and increase the number of potential reads per sample and our assay is enabled for high-throughput and automated formats. We developed a multiplexed targeted amplification NGS bisulfite mapping assay, that measures the state of methylation of regions spanning 100 to 200 bp around the “HCC-spec” and “HCC-detect” CpGs in up to 200 people in parallel. We had only 5 cases out of the 402 that didn’t provide sufficient reads (a threshold of 100 reads per gene) in at least one of genes. The average coverage per sample for each of the gene regions ranged from 3160 to 5181, suggesting that most plasma samples from either healthy controls or cases have sufficient DNA to generate methylation information on these genes.

In difference from examining DNA methylation in a tumor biopsy where a significant fraction of DNA is derived from the tumor (as in the TCGA methylation database), tumor DNA in plasma is mixed with DNA from potential other sources and the extent of dilution of tumor DNA in other DNA is unknown. Thus, the level of methylation of plasma DNA might reflect an unpredictable dilution of tumor DNA; the level of methylation of cfDNA is therefore a function of the state of methylation of DNA in the tumor and the unknown and stochastic mixture with other DNA. Thus, it is anticipated that the level of methylation is lower than what we derived from examining tumor DNA methylation data. However, if the methylation profile of the tumor DNA is categorically distinct from the methylation profile of other potential sources of DNA in plasma as anticipated by the analyses above (Figs. 1–3), we expected that it would be detectable even on a high background. We used targeted NGS, which provides DNA methylation profiles at a single DNA molecule resolution.

All 5 CpG positions included in “HCC-detect” and “HCC-spec” are significantly more methylated than in either healthy controls, chronic hepatitis B or both or non-HCC cancers (*CCNJ* is nominally significant when compared to healthy controls and significant against chronic hepatitis B). As expected, most control samples are completely unmethylated with a median methylation of less than 0.5%. Methylation in HCC is heterogeneous ranging from baseline levels to 80% (Fig. 5A). This probably reflects to some extent random heterogeneous mixture of tumor and normal cfDNA in plasma but also the heterogeneity of methylation of these sites in the original tumor (Figs. 1 and 2). These data confirmed that the “HCC-detect” and “HCC-spec” CpGs selected using TCGA tumor DNA methylation data are differentially methylated in HCC patients’ plasma in a second independent clinical cohort from a different geographic setting using a completely different methylation mapping method.

Our markers are CpG-rich and by sequencing a 100-200 bp region proximal to the CpG sites that were discovered in the Illumina 450 K array data we capture information on the state of methylation of several neighboring CpGs to the CpGs that were selected using the TCGA data (Supplementary Fig. 3C). Our data indicates that in HCC samples the levels of methylation across CpGs in each of the five sequenced regions are correlated while only sporadic methylation is detected in the healthy controls which might be biological or just a result of spurious infrequent errors in bisulfite conversion or sequencing. By examining the entire profile, we can increase our specificity and exclude such cases. To reflect the consistency of methylation across the region we calculated the medians for each CpG region (as is visualized in heatmap in Fig. 5B) The medians for each CpG region (Fig. 5B) and the “HCC-detect” sum (Fig. 5B) clearly separate the HCC samples from chronic hepatitis B and healthy controls (Fig. 5B). Since our training set analysis suggested that methylation in any one of the four CpGs is sufficient to classify a sample as HCC we have given equal weight to each region and used the normalized sum of the medians as the “HCC-detect” M score and the normalized median for *F12* region as the “HCC-spec” M score. Logistic regression of M scores calculated (Fig. 6B, E) the predicted probability of HCC for each sample; 89% of the HCC samples scored close to 1 at all stages in contrast to 4% of the healthy controls which were misclassified as HCC (Fig. 6C, Supplementary Table 6). A combined “HCC-detect” and “HCC-spec” score which accurately classified predicted probability had an ROC of 0.94 (Fig. 7A), (Fig. 2D and E) classified HCC from other cancers in the TCGA data, classified 256 out of the 302 HCC samples as HCC (86%) and misclassified 2 out of 46 healthy controls as HCC (Fig. 7C, D, E). We validated the “HCC specificity” of our approach by examining an independent cohort of healthy people plasma and plasma from patients with non-HCC tumors, in both cohorts our assay detected in very low methylation scores an HCC prediction (Figs. 5, 6 and 7). Our results validated the “HCC-detect” and “HCC-spec” markers, in cfDNA clinical samples and provided a way forward for developing a feasible, high-throughput and low-cost test for noninvasive screening and detection of HCC that is tumor specific. We termed the combined “HCC-detect” and “HCC-spec” “epiLiver”. The robustness of these markers was validated at several levels in tumors and plasma DNA using different platforms and different cohorts from geographically distinct area. The robustness of the markers in the clinical study in Bangladesh is supported by bootstrapping and “cross-validation” analyses.

The sensitivity of the CF plasma DNA test was lower than in the TCGA data 85–89% in the clinical study compared to 96 to 98% in the analysis of TCGA DNA methylation values. There are a few possible reasons. First, TCGA data is derived from tumor tissue, while cfDNA that originates from tumors is mixed with DNA from blood and other normal tissues in unpredictable ratios. Second, the amount of cfDNA varies across patients. Third, quality of plasma derived in clinical settings is probably not even and different levels of mixture of genomic DNA might be caused by different handling of samples. Fourth, different genomic regions might be heterogeneously represented in cfDNA in different samples. Nevertheless, our assay demonstrates high sensitivity and specificity as well as high-throughput. One of the challenges of early noninvasive tumor detection is defining the tissue of origin. Remarkably only one CpG was sufficient to accurately reveal the tissue of origin suggesting that an economical screen for specific tumor types might be feasible and that more complex tests that are based on multiple DNA methylation sites and regions might not be necessary. This was validated against a large cohort of publicly available DNA methylation data in TCGA, a different publicly available dataset from both tumor and plasma as well as by “cross validation” in the clinical study in Bangladesh. However, full assessment of the clinical value of the “HCC-detect” and “HCC-spec” markers for early detection of HCC requires an adequately powered prospective study.

Although the 5 regions included in the “HCC-spec” and “HCC-detect” are devoid of methylation in the vast majority of healthy controls as is the case in the TCGA data, a small number of healthy normal individuals (2 in the combined “HCC-detect” and “HCC-spec” score) had a profile that resembled HCC patients with consistent methylation across all CpGs. At this stage we don’t know whether these false positives are truly “false” or whether they represent undetected cases. Our clinical study did not include follow up of such cases. One of the challenges of early detection is to further study and understand these “false positives” as well as deciphering the exact boundary between healthy and controls, lowering the threshold will increase the sensitivity of detection but this should be done without increasing the level of “false discoveries”. Although the vast majority of hepatitis B patients had very low level of methylation and there is no statistically significant difference noted between healthy controls and CHB patients mean M score, there was a higher fraction of HCC classifications in the chronic hepatitis B group (12%). The fact that we got a higher fraction of HCC calls in the HepB group might be consistent with the higher risk of conversion to HepB; follow up of these cases is warranted.

We compared our epiLiver test to AFP a standard biomarker for HCC. The effectiveness of AFP for screening has been disputed^5^. We show that in this study AFP had high specificity and reasonable sensitivity (Fig. 8A, D) that was lower than epiLiver (Fig. 8E, F). However, a combination of AFP seems to increase detection at earlier stages (Fig. 8C, G). However, this needs to be tested in a larger study.

One of the biggest advantages of “epiLiver” is its specificity for HCC, particularly, using “HCC-spec” which allows accurate detection of the disease. Additionally, the use of NGS high-throughput, and cost-effective approach. Limiting the number of regions assayed we can increase the average read per region and reduce the cost.

A limitation of our study is that the mean age of healthy controls, hepatitis B and HCC patients are significantly different and that the study was limited to people from Bangladesh. The study should be repeated in people from other ethnicities. Nevertheless, it should be noted that the markers were selected and validated for specificity to HCC in TCGA database, which includes data from different ethnicities and our analysis shows that the methylation state of these markers is not significantly different between different ethnicities (Supplementary Fig. 2).

In summary, our study reveals a feasible and accurate noninvasive test for detection of HCC. Applying this test for screening people at risk for developing HCC could potentially have an important impact on relieving the burden of this tumor on the individual as well as the health system.

## Methods

This research complies with all relevant ethical regulations. The study protocol was approved by IRB board of ICDDR,B (Dhaka, Bangladesh).

### DNA methylation public data

Normalized DNA methylation beta values for 450 K sites included in Illumina DNA methylation arrays for a total of 11,636 samples from healthy blood, normal tissues, cancer tissues and noncancer-associated tissues NAT were downloaded from TCGA and GEO sites as listed in Table 1.

We first generated a list of 47981 CpG positions that were hypomethylated in 17 different somatic tissues (beta = <0.1 and median <0.02) using Illumina 450 K array data in GSE50192. We then generated a list of 68260 unmethylated CpG positions in blood DNA in each of the 312 individuals in GSE61496. To increase the robustness of the list and to exclude sites with residual variation in methylation across individuals that are derived from sex or age differences, we overlapped this list with a list of 55959 of unmethylated CpGs in blood DNA in all 656 individuals, males and females aged from 19 to 101 years (GSE40279). We got the list of 41622 CpGs of unmethylated CpGs in blood DNA from both dataset GSE61496 and GSE40279 in all 968 individuals.

We then overlapped the two lists (unmethylated in blood and in tissue) to obtain a list of 28,775 CpGs that are unmethylated in every single individual in both blood datasets and 17 somatic tissues.

### Discovery and validation of “HCC-detect” classifier and polygenic methylation score computation

We compared the state of methylation of the list of 28,775 CpG sites that are ubiquitously “methylation-resistant” in normal tissues, between HCC (GSE54503; *n* = 66) and non-HCC liver samples including fibrosis and cirrhosis (GSE61258; *n* = 79). Significant methylation differences between HCC and control liver DNA methylation were computed using t statistics adjusted by Bonferroni corrections for 450 K multiple tests.

A list of the top 20 CpG sites (delta > +/−0.2) was further reduced by penalized regression. A weighted polygenic methylation score for HCC was computed by a multivariable linear regression equation based on methylation values for these four CpG positions in the training data (Supplementary Table 9). “HCC-detect” = [(β1 × 0.75) + (β2 × 0.43) + (β3 × 0.64) + (β4 × 0.8) + 0.064]. β1: methylation level of *VASH2*; β2: methylation level of *GRID2IP*; β3: methylation level of CHFR; β4: methylation level of *CCNJ*.

“HCC-detect” was validated by computing the “HCC-detect” methylation scores in DNA methylation 450 K array data for 739 HCC samples (GSE76269, GSE75041, GSE54503 and TCGA), 116 adjacent non-tumor tissues (NATs)

(GSE54503 and TCGA), 16 healthy liver (GSE69852, GSE76269) and 968 blood samples (GSE40279, GSE61496). The “HCC-detect” score was then computed using the above equation in 450 K DNA methylation array data for healthy blood (GSE40279, GSE61496; *n* = 968), healthy liver tissue or adjacent non-tumor tissues (GSE76269 and TCGA; *n* = 66), other liver disease including cirrhosis and fibrosis (GSE61258, *n* = 79), and liver cirrhosis patients (GSE157973, *n* = 130), other healthy tissues (GSE63704, GSE65821, GSE85464, GSE85566, GSE60185, GSE53051, GSE52955 and GSE42752, *n* = 228), non-liver adjacent non-tumor tissues (TCGA, *n* = 677), cancers from 31 other origins (TCGA, *n* = 8961) and 739 liver cancer samples (GSE76269, GSE75041, GSE54503 and TCGA), total *n* = 11718 (Table 1 and Supplementary Table 1).

### Discovery and validation of “HCC-spec”

A discovery cohort of 230 randomly selected DNA methylation samples from TCGA representing 17 different cancers (210 samples), 10 HCC samples and 10 healthy blood samples was used to calculate the difference average methylation at each of the 450 K CpG positions between HCC and all other groups. We didn’t limit our search to the 28,775 methylation-resistant CpGs, in order not to miss liver-specific methylated CpGs that are methylated in both normal and cancer liver tissue. 7 CpGs were shortlisted (delta >0.5 and Bonferroni adjusted *p* value of Q < 10^−20^). A multivariable linear regression with the 7 CpGs as co-variates revealed that cg14126493 at the body of the *F12* gene has the largest effect. A weighted methylation score for *F12* computed by a linear regression equation (Supplementary Table 3). “HCC-spec” = [(β1 × 1.35) + 0.18]. β1: methylation level of cg14126493. For validation, the weighted methylation score was computed for the entire data base of 11918 450 K arrays.

Further validation was performed om GSE63775 (Wen et al.^28^) a data base of cfDNA and tumor tissue as well as NAT-HCC DNA methylation data and plasma from liver cirrhosis and normal patients derived by bisulfite conversion followed by methylated CpG tandem amplification and sequencing which enriches for methylated CpGs (*n* = 191). The level of methylation is expressed as the count of methylated reads as explained in Wen et al.^28^ The count of methylated reads in genomic regions containing each of the 5 CpGs of the “HCC-detect” and “HCC-spec” markers were compared between two groups in 57 tissue samples and 94 plasma samples.

We used the Integrative Genomics Viewer (IGV) browser and the dbSNP database (version 1.4.7) to identify SNPs in our DNA sequence data and verify that these SNPs were not present at any of the highly-specific methylation sites within HCC-detect and HCC-spec. Moreover, all the CpG sites identified are observed in almost every participant in a partially methylated form (both C and T forms are present) and these are almost never 50, 100% as expected for SNPs and are rarely 0%. During this process, we identified a single SNP in one of the CpG sites associated with the *F12* gene (rs761368173). This SNP is a frameshift deletion (-/C) mutation with allele frequencies of 0.000009 and 0.999991.

### Power calculations for clinical study

To estimate the minimum sample size required to validate our findings in a clinical study we performed power analyses. A power calculation using the pooled standard deviation of the methylation scores (sigma) for the healthy blood and HCC tissues (0.31) and desired power of 0.8 shows that a sample size of 40 for each group is required to detect a delta beta of 0.2 between cancer and control. We performed a power analysis on the cfDNA plasma methylation data from the GSE63775 study for each gene region separately. The power calculation utilized the combined standard deviation of *CCNJ* (spooled =7.96), F12 (spooled =44.8), *VASH2* (spooled =30.2), *GRID2IP* (spooled =0.55), and *CHFR* (spooled =0.56) in normal and HCC plasma, with the aim of achieving a power of 0.8. The results indicate that a sample size of 23 for *CCNJ*, 8 for *F12*, 17 for *VASH2*, 142 for *GRID2IP*, and smaller than 10 for *CHFR* was sufficient to detect a difference of 6.77, 63, 29.3, 0.19 and 0.44 reads between groups, respectively. We reasoned that a samples size of 400 would be sufficiently powered to detect significant differences in methylation between HCC and control even in *GRID2IP*.

### Clinical study design

The participants received compensation to be part of the study. Study protocol was approved by IRB board of ICDDR,B (Dhaka, Bangladesh) study protocol PR-18025. 402 participants were recruited from the Dhaka area to the study included 49 healthy controls, 51 Chronic hepatitis B patients, 102 non-HCC patients and 302 HCC patients from stages 0 to D (HCC 0 *n* = 2, HCCA *n* = 32, HCC B *n* = 86, HCC C *n* = 106, HCC D *n* = 76 (See Table 2 for demographics). We next recruited additional 102 non-HCC patients (Supplementary Table 10) to test the performance of “HCC-spec” (*F12*) marker that was selected to separate HCC from other cancer types as well as 50 plasma samples that were derived from healthy people and were procured from Innovative^TM^ Research (see Table 2 for clinical parameters). The age of the healthy control and the chronic Hepatitis B (CHB) groups were significantly younger than the HCC group, however there was no significant effect of age on HCC prediction by DNA methylation as determined by a logistic regression in either the HCC or control groups. However, in the CHB group age had a significant effect on HCC prediction by the DNA methylation test, consistent with the idea of a higher probability for older patients with chronic hepatitis to convert to HCC. There were no differences between the groups in the sex distribution. However, there was a somewhat significant lower alcohol use and higher fraction of smokers in the HCC groups (Table 2). HCC staging was diagnosed according to EASL–EORTC Clinical Practice Guidelines: Management of hepatocellular carcinoma^43^. Hepatitis B diagnosis was confirmed using AASLD practice guideline for chronic Hepatitis B (https://www.aasld.org/publications/practice-guidelines). All participants were properly informed about the study and have signed the informed consent form approved by the ICDDR,B IRB. Inclusions criteria were participants of either sex 18 to 70 years of age, confirmed diagnosis of HCC using EASL-EORTC guidelines and chronic hepatitis B using AASLD guidelines, non-metastatic liver cancer, Hepatitis B surface antigen positive by ELISA and persistence of > 6 months. AFP levels in plasma were measured using a Chemiluminescence Immunoassay as part of the standard procedure during patient visits.

**Table 2:** Demographics of study participants

	N	Age		Sex		Alcohol		Smoking		AFP	
		Mean	Significance	Male/Female	Significance	Fraction users	Significance	Fraction users	Significance	N	Mean ± St error
Control	49	26.14		42/7	0.99	0.2		0.32		1	1.02 + /− NA
CHB	51	28.71	0.99	42/9	0.99	0.21	0.99	0.35	0.99	22	2.2 + /− 0.9
HCC 0	2	62	NA	2/0	0.99	NA	NA	NA	NA	2	1387.4 + /− 866.4
HCC A	32	49.15	<0.0002	27/5	0.99	0.088	0.35	0.64	0.02	30	6460.5 + /− 12192.3
HCC B	86	49.12	<0.0003	72/14	0.99	0.069	0.047	0.52	0.13	79	17246.3 + /− 44977.5
HCC C	106	49.34	<0.0004	85/21	0.99	0.037	0.0047	0.54	0.05	100	11968.4 + /− 31972.1
HCC D	76	52.45	<0.0005	64/12	0.99	0.064	0.042	0.66	0.001	71	29499.9 + /− 67562.3
NON-HCC Cancers	102	47.14	NA	48/54	NA	0.02	NA	0.39	NA	NA	NA
Healthy Plasma (Innovative^TM^ Research)	50	38.1	NA	50/0	NA	NA	NA	NA	NA	NA	NA

Significant difference from the control group was tested by Kruskal-Wallis non-parametric one-way ANOVA and was adjusted by Dunn’s multiple comparisons test. The two HCC stage 0 samples were included for statistics in the HCC A group. (NA-not available).

AFP values are given in ng/mL.

Exclusion criteria were unwilling or unable to provide informed consent, unwilling or unable to comply with requirements of protocol, participation in a different clinical trial investigating a vaccine, drug, medical device or medicinal procedure less than 4 weeks preceding the current study, planned participation in another clinical trial during present study period, known case of cirrhosis, any other known inflammatory disease (bacterial or viral infection with the exception of hepatitis B or C), known case of diabetes, asthma, autoimmune disease, any other diagnosed cancer, for healthy controls any known inflammatory or infectious disease including Hepatitis B and Hepatitis C and any diagnosis of chronic disease, cancer medication use or drugs of abuse. Blood samples from non-HCC patients were similarly derived at ICDDR,B exclusion criteria were diagnosis of any liver disease, diagnosis of HCC or liver metastasis of other cancers, inclusion criteria were cancer of one of ten common cancers: bladder cancer, breast cancer, cervical cancer, head and neck squamous carcinoma, lung cancer, colon cancer, esophageal carcinoma, ovarian cancer, prostate cancer, gastric cancer, gall bladder cancer, renal cell carcinoma, thyroid cancer, and soft tissue sarcoma. Cancers were diagnosed according to ESMO-Clinical Practice Guidelines. The stage was determined according to the American Joint Committee on Cancer (AJCC)/Union for International Cancer Control (UICC) TNM staging system (7th edition) (see Supplementary Table 6 for clinical parameters). Patients were assigned an ID that was kept confidential according to hospital regulations and identity was revealed only to approved hospital personnel. The participants received compensation to be part of the study.

Participants provided consent for DNA methylation biomarker research. Blood sample collection ad plasma separation was performed at ICDDR,B in Dhaka Bangladesh and was then shipped to HKG epitherapeutics for further analysis. The HKG epitherapeutics lab team was blinded on the identity of the samples throughout the lab analytic procedures. Data was then analyzed in Montreal and shared with ICDDR,B who provided the results to the respective clinical personnel.

### Preparation of cfDNA plasma

Blood was collected in 9-ml tubes containing K3-EDTA and processed within 1 h. Plasma and peripheral blood monocyte separation was performed according to GE Healthcare Cat No 71 = 7167-00 protocol. Plasma was frozen and shipped. Plasma samples (1 ml) were thawed, and DNA was extracted by previously described guanidine isothiocyanate method^44^ and binding to silica magnetic beads followed by 80% ethanol washes and water elution.

### Multiplexed targeted DNA methylation sequencing with Illumina NGS

Bisulfate conversion was performed using EZ-96 DNA Methylation MagPrep (D5041, Zymo Research) followed by two rounds of polymerase chain reaction. For the first round we used primers that included an anchoring sequence and sequences targeting a region of 100 to 200 bp that included cg02012576 (CHFR), cg03768777 (*VASH2*), cg05739190 (*CCNJ*), cg24804544 (*GRID2IP*) and cg14126493 (*F12*) using Bio-Rad C1000 Touch Thermal Cycler (Bio-Rad Laboratories, CA, USA) (the primers are available upon request). 5 microliters of the first PCR reaction were subjected to a second round of PCR amplification using primers containing indexes for barcoding the samples (the primers are available upon request). PCR products were pooled, and the pooled library was then purified twice using AMPure XP Beads (Beckman Coulter Life Sciences, CA, USA) and quantified by RealTime PCR using NEBNext® Library Quant Kit for Illumina (New England Biolabs, MA, USA). Barcoded libraries from all samples were sequenced on the Illumina platform using MiSeq Reagent Nano Kit V2 using a 250 × 2 paired-end sequencing protocol (Illumina, CA, USA).

For our targeted bisulfite sequencing experiments, we utilized Trim-galore (parameters: trim_galore –illumina –paired –fastqc) (https://zenodo.org/record/5127899#.Y7RxfOzMJqs) to trim sequencing adapters and low-quality data from the raw, paired-end reads and obtain clean data for subsequent analysis. We then aligned the clean data to five reference genomes cg02012576 (CHFR), cg03768777 (*VASH2*), cg05739190 (*CCNJ*), cg24804544 (*GRID2IP*), and cg14126493 (*F12*) using Bismark (https://www.ncbi.nlm.nih.gov/pmc/articles/PMC3102221/) and deduplicated the reads using the UMIs in the forward primers to reduce the PCR amplification bias (deduplicate_bismark --paired --barcode –bam). Using Bismark methylation extractor (bismark_methylation_extractor --p --bedGraph --counts --scaffolds --no_overlap), we calculated the methylation level at each CpG site by extracting methylation information from the aligned reads. We set the threshold per gene to be included in methylation analysis at 100 reads. 5 samples with sequencing reads below 100 were removed from the analysis and we remained with 397 informative samples in the Bangladesh cohort.

### Calculation of M scores and probability scores for predicting HCC in cancer patients

We used median rather than average to exclude situations where a high average is driven by a spurious high methylation of a single CpG. Median values of percentage methylation from 0 to 100 were normalized (log 2) and a “HCC-detect” M score was computed from the SUM of normalized medians of CHFR, *VASH2*, *CCNJ* and *GRID2IP* regions giving equal weight to each region. Similarly, the “HCC-spec” M score detecting HCC specificity versus other cancers was computed from the median methylation of the *F12* region. We used logistic regression in Prism to model M score as a predictor of probability of HCC and computed a predicted probability for each person using the logistic regression equation.

### AFP-based prediction of HCC in cancer patients

AFP levels in plasma were determined by ELISA using the Chemiluminescence Immunoassay. Out of 302 HCC patients, 282 had AFP data and 20 were missing (Table 2). As control, we used AFP data from 22 CHB and 1 healthy individual (Table 2). We used the commonly used threshold of 400 ng/mL for AFP levels to identify possible cases of HCC. This threshold has been described in recent publication that reviewed 29,828 articles and included 59 studies and 1 review with a total of 11,731 HCC-confirmed patients and 21,972 control cases without HCC. A sample was scored as HCC if AFP > 400 mg/ml. The threshold for epiLiver or the sum of probabilities of “HCC-detect” and “HCC-spec” was set at 1.62. We compared the performance of epiLiver and AFP at the above threshold on the same patients and controls with available AFP data, accounting for the missing data (https://pubmed.ncbi.nlm.nih.gov/32053643/). For a combined AFP and epiLiver predictor a sample was scored as HCC if either AFP or epiLiver prediction was HCC.

### Computational and statistical methods used in the study

We used the computing environment R version 3.4.4. For penalized regressions we used the R statistical package “penalized” (*44*), for multivariable linear regression analysis we used the lm function in R to fit linear models and for genomic feature enrichment we performed a hypergeometric test using “phyper” function in R. For other statistical analyses we used Graph Pad Prism 9.01 statistical package. Normality and log normality were tested using Shapiro-Wilk and Kolmogorov-Smirnov tests. Nonparametric statistics were used to test significance when data failed normality tests. For two group comparisons a two tailed Mann Whitney test was used and for multiple group comparisons we used Kruskal-Wallis test followed by Dunn’s multiple comparisons test to derive the adjusted p value. ROC was computed using the ROC test in GraphPad. To generate heatmaps we used GENE E software from the Broad institute (https://software.broadinstitute.org/GENE-E/).

For the comparison of means between normal and cancer groups for MScore_detect and MScore_spec biomarkers, the t-test was performed using the bootstrap resampling method with 1000 samples. Confidence intervals and p-values were calculated using the percentile method. The code uses pandas, numpy, sklearn.utils (for resample function), and scipy.stats (for ttest_ind function) (10.5281/zenodo.7820166). Cross-validation was performed using “tidyverse” and “caret” R packages (10.5281/zenodo.7823332) with the validation set approach, which consists of randomly splitting the data (46 healthy controls, 397 cancer individuals with stages A to D and 50 healthy plasmas from a different cohort) to training and validation datasets. We ran the cross-validation three times with the training data including 50% of the entire dataset. We computed model performance metrics (Supplementary Table 6). Then, using the models obtained from three randomly sample training dataset, we examined, how well does the model predict cancer in the remaining validation dataset (Supplementary Fig. 4).

### Reporting summary

Further information on research design is available in the Nature Portfolio Reporting Summary linked to this article.

## Supplementary information

Supplementary Information

Reporting Summary

## Data Availability

The raw multiplexed targeted DNA methylation sequencing data supporting the findings of this study are available from the Sequence Read Archive (SRA) PRJNA962995 under controlled access due to their commercial value. The data consist of fastq files generated from MiSeq sequencing. To obtain the data, a material transfer agreement (MTA) is required, and access is limited to academic use only. Access requests will receive a response within approximately a week from the date of the request. Once access has been granted, data will be kept available for the requester for a period of 30 days, with the possibility to accommodate specific needs whenever possible. For further information and data requests, please contact the corresponding author, David Cheishvili, at david.cheishvili@hkgepitherapeutics.com. Source data are provided with this paper.

## Source data

Source Data

## Code availability

The Python code for bootstrapping analysis can be downloaded from Zenodo (10.5281/zenodo.7820166), the R code for cross-validation can be downloaded from Zenodo (10.5281/zenodo.7823332), and the Python script for generating a confusion matrix heatmap can be downloaded from Zenodo (10.5281/zenodo.7823382).
