# Supplementary material for: A high-throughput test enables specific detection of hepatocellular carcinoma
Source: Nat Commun. 2023 Jun 7;14:3306. doi: 10.1038/s41467-023-39055-7 (PMC10247794; doi:10.1038/s41467-023-39055-7)
Supplement: Supplementary file 1 — Supplementary Information [file 41467_2023_39055_MOESM1_ESM.pdf]

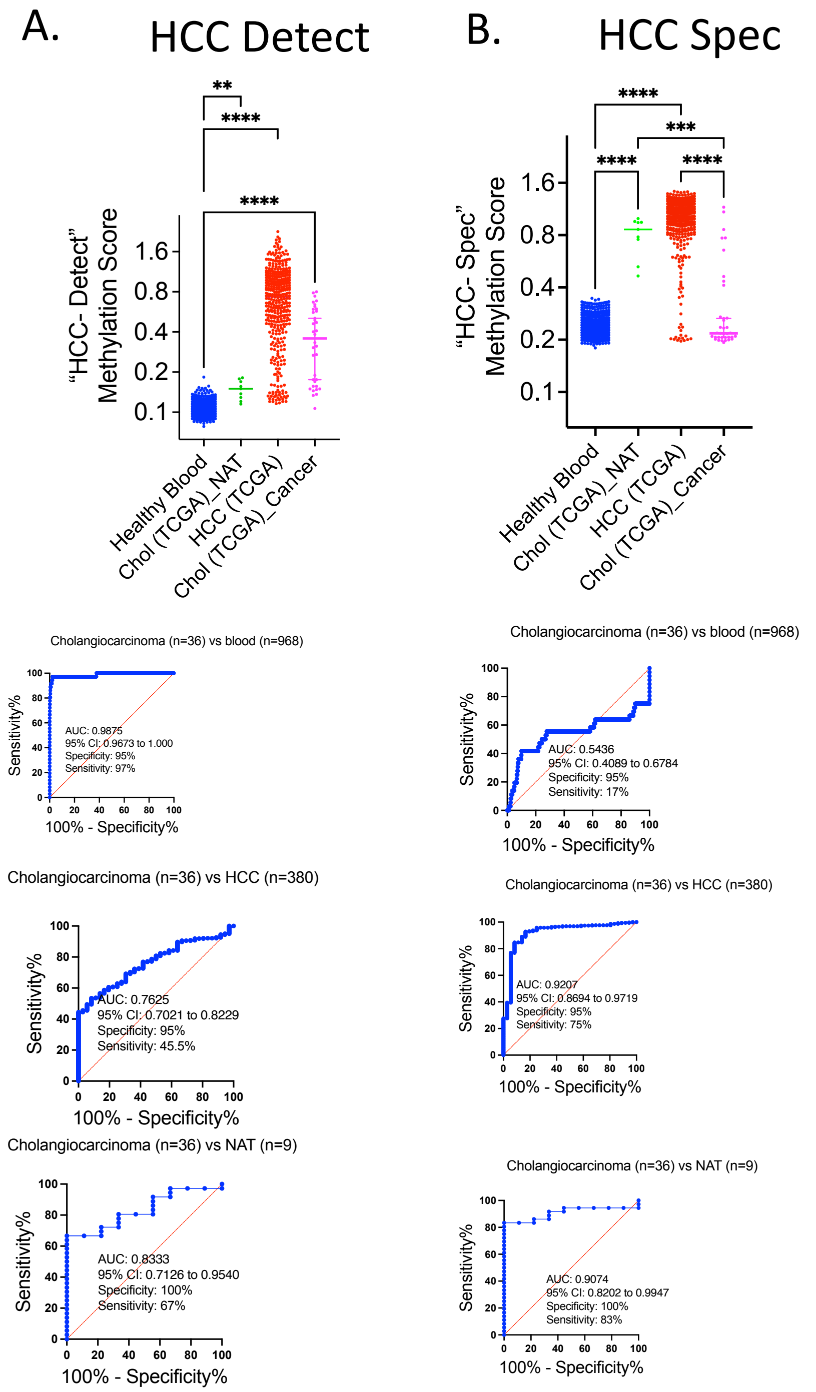
A.
HCC Detect
**
****
****
"HCC- Detect" Methylation Score
1.6
0.8
0.4
0.2
0.1
Healthy Blood
Chol (TCGA)_NAT
HCC (TCGA)
Chol (TCGA)_Cancer
B.
HCC Spec
****
***
****
****
"HCC- Spec" Methylation Score
1.6
0.8
0.4
0.2
0.1
Healthy Blood
Chol (TCGA)_NAT
HCC (TCGA)
Chol (TCGA)_Cancer
Cholangiocarcinoma (n=36) vs blood (n=968)
Sensitivity%
100% - Specificity%
AUC: 0.9875
95% CI: 0.9673 to 1.000
Specificity: 95%
Sensitivity: 97%
Cholangiocarcinoma (n=36) vs blood (n=968)
AUC: 0.5436
95% CI: 0.4089 to 0.6784
Specificity: 95%
Sensitivity: 17%
Cholangiocarcinoma (n=36) vs HCC (n=380)
AUC: 0.7625
95% CI: 0.7021 to 0.8229
Specificity: 95%
Sensitivity: 45.5%
Cholangiocarcinoma (n=36) vs HCC (n=380)
AUC: 0.9207
95% CI: 0.8694 to 0.9719
Specificity: 95%
Sensitivity: 75%
Cholangiocarcinoma (n=36) vs NAT (n=9)
AUC: 0.8333
95% CI: 0.7126 to 0.9540
Specificity: 100%
Sensitivity: 67%
Cholangiocarcinoma (n=36) vs NAT (n=9)
AUC: 0.9074
95% CI: 0.8202 to 0.9947
Specificity: 100%
Sensitivity: 83%

Supplementary Figure 1. Differential methylation of "HCC-detect" and "HCC-spec" CpGs in healthy blood, NAT, HCC and intrahepatic cholangiocarcinoma.

Scatterplot of the "HCC-detect" (A, upper panel) and "HCC-spec" (B-upper panel) for each of the samples in the healthy blood (n=968), NAT (TCGA, n=9) and intrahepatic Cholangiocarcinoma (TCGA, n=36) and HCC (TCGA, n=380) groups. ROC curve of "HCC-detect" (A, lower panel) and "HCC-spec" (B, lower panel) in healthy blood, NAT, and intrahepatic cholangiocarcinoma. Figure A and B shows scatterplots for comparisons with a P value of 0.05 or less, indicating that differences between groups are only considered significant if they meet this threshold. Any comparisons with a higher P value are not displayed. Kruskal-Wallis nonparametric one-way ANOVA with Dunn's multiple comparisons test was used to compare the groups. The line at the median with 95% confidence interval is shown in the plot. Source data are provided as a Source Data file.

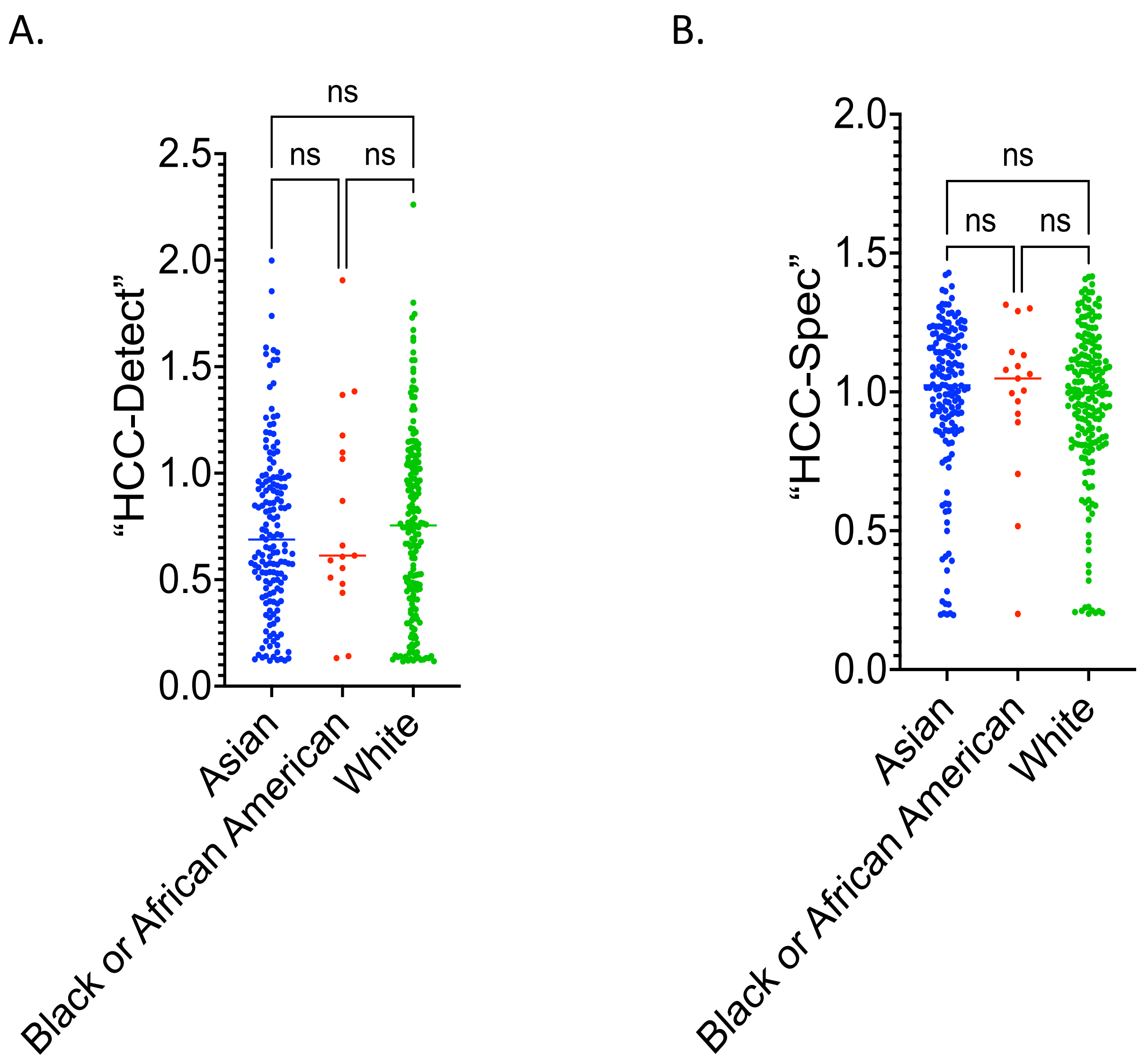

A.
ns
ns
ns
2.5
2.0
1.5
1.0
0.5
0.0
“HCC-Detect”
Asian
Black or African American
White
B.
2.0
1.5
1.0
0.5
0.0
ns
ns
ns
“HCC-Spec”
Asian
Black or African American
White

Supplementary Figure 2. Scatterplot of the "HCC-detect" (A) and "HCC-spec" (B) for each of the samples in the HCC Asian, black or African American and white people (TCGA, n=365). Kruskal-Wallis nonparametric one-way ANOVA with Dunn's multiple comparisons test was used to compare the groups. Source data are provided as a Source Data file.

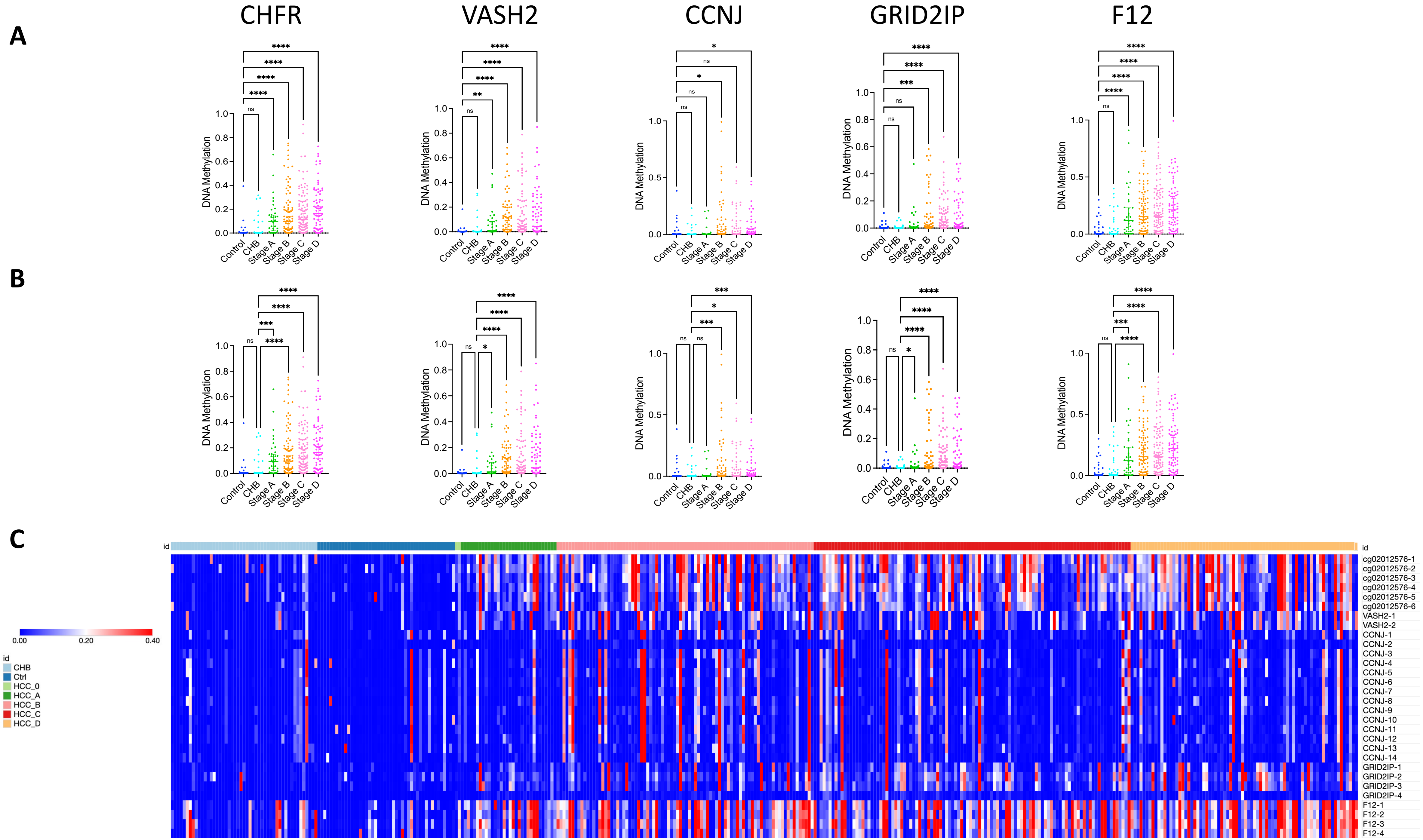
A
B
C
CHFR
VASH2
CCNJ
GRID2IP
F12
DNA Methylation
Control
CHB
Stage A
Stage B
Stage C
Stage D
ns
*
**
***
****
id
0.00
0.20
0.40
CHB
Ctrl
HCC_0
HCC_A
HCC_B
HCC_C
HCC_D
cg02012576-1
cg02012576-2
cg02012576-3
cg02012576-4
cg02012576-5
cg02012576-6
VASH2-1
VASH2-2
CCNJ-1
CCNJ-2
CCNJ-3
CCNJ-4
CCNJ-5
CCNJ-6
CCNJ-7
CCNJ-8
CCNJ-9
CCNJ-10
CCNJ-11
CCNJ-12
CCNJ-13
CCNJ-14
GRID2IP-1
GRID2IP-2
GRID2IP-3
GRID2IP-4
F12-1
F12-2
F12-3
F12-4

Supplementary Figure 3. Differential methylation of "HCC-detect" and "HCC-spec" CpGs at different stages of HCC in the Dhaka clinical study. Median methylation and 95% confidence interval for the four CpGs included in the "HCC-detect" set (cg02012576 (CHFR), cg03768777 (*VASH2*), cg05739190 (*CCNJ*), cg24804544 (*GRID2IP*) and "HCC-spec" cg14126493 (*F12*) with ANOVA analysis of control samples vs CHB and the four stages of cancers (n=46 for healthy controls, n=49 for CHB, Stage A, n=34, Stage B, n=86, Stage C, n=106 and Stage D, n=76. (A) and CHB vs Control and the four stages of cancers (B) (n for each group as indicated in Fig. 5). C. Heatmap illustrates the methylation values for all the CpGs included in the sequenced regions for all five genes. Figures And B show scatterplots for comparisons with a P value of 0.05 or less, indicating that differences between groups are only considered significant if they meet this threshold. Any comparisons with a higher P value are not displayed. The line at the median with 95% confidence interval is shown in the plot.

Source data are provided as a Source Data file.

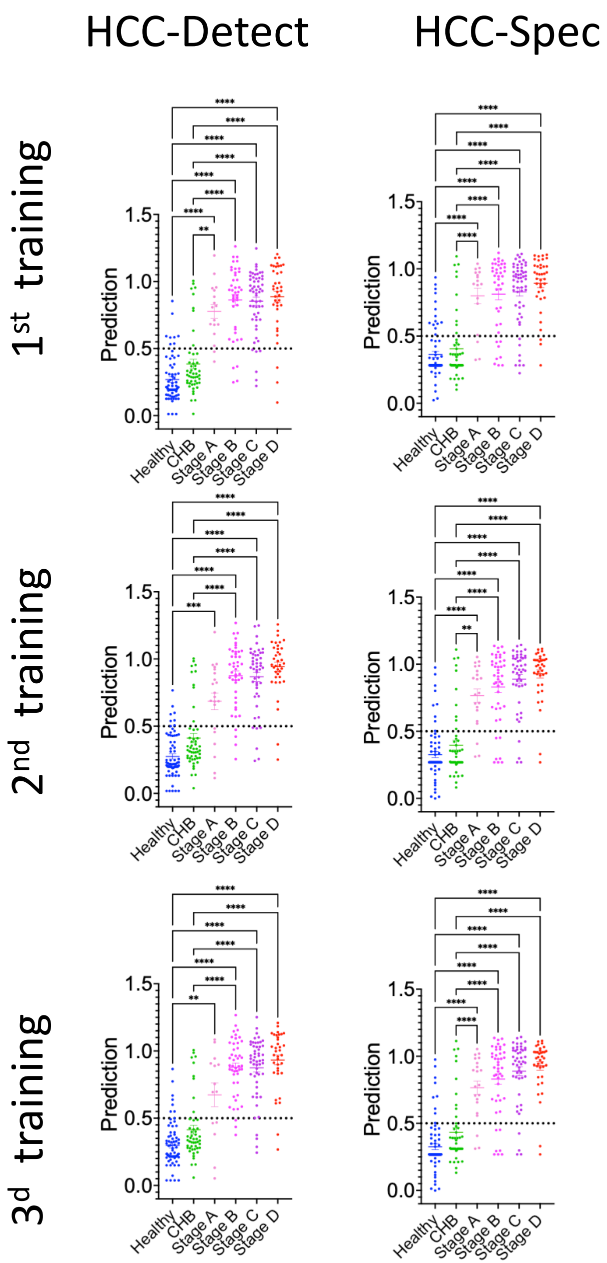
HCC-Detect
HCC-Spec
1st training
2nd training
3d training
Prediction
0.0
0.5
1.0
1.5
Healthy
CHB
Stage A
Stage B
Stage C
Stage D
****
***
**

Supplementary Figure 4. Prediction of HCC using three models which were trained on three randomly selected training subsets in randomly selected validation subset. The charts show HCC prediction (0 and 1) for each model in plasma cfDNA samples from different stages of HCC, nonHCC cancers, CHB and nonaffected controls. Figure shows scatterplots for comparisons with a P value of 0.05 or less, indicating that differences between groups are only considered significant if they meet this threshold. Any comparisons with a higher P value are not displayed.

Significance was determined by Kruskal-Wallis nonparametric one-way ANOVA with Dunn's multiple comparisons test (**** $p<0.0001$, *** $p<0.001$, ** $p<0.01$, *$p<0.05$, n.s. nonsignificant). Sample sizes after cross-validation were n=70 for healthy controls, n=49 for CHB, Stage A, n=17, Stage B, n=41, Stage C, n=57 and Stage D, n=38 (the sample sizes were obtained after splitting the cohort into validation and training sets.). The line at the median with 95% confidence interval is shown in the plot. Source data are provided as a Source Data file.

Supplementary Table S1 Cancer and NAT samples analyzed from TCGA

Study Abbreviation	Study Name	Cancer Subject number	NAT subject number	Total
ACC	Adrenocortical carcinoma	80	0	80
BLCA	Bladder Urothelial Carcinoma	417	21	438
BRCA	Breast invasive carcinoma	796	95	891
CESC	Cervical squamous cell carcinoma and endocervical adenocarcinoma	309	3	312
CHOL	Cholangiocarcinoma	36	9	45
COAD	Colon adenocarcinoma	414	38	452
ESCA	Esophageal carcinoma	186	16	202
GBM	Glioblastoma multiforme	153	2	155
HNSC	Head and Neck squamous cell carcinoma	530	50	580
KICH	Kidney Chromophobe	66	0	66
KIRC	Kidney renal clear cell carcinoma	325	159	484
KIRP	Kidney renal papillary cell carcinoma	276	45	321
LAML	Acute Myeloid Leukemia	140	0	140
LGG	Brain Lower Grade Glioma	534	0	534
LIHC	Liver hepatocellular carcinoma	380	50	430
LUAD	Lung adenocarcinoma	411	22	433
LUSC	Lung squamous cell carcinoma	337	36	373
MESO	Mesothelioma	87	0	87
OV	Ovarian serous cystadenocarcinoma	10	0	10
PAAD	Pancreatic adenocarcinoma	185	10	195
PCPG	Pheochromocytoma and Paraganglioma	184	3	187
PRAD	Prostate adenocarcinoma	503	50	553
READ	Rectum adenocarcinoma	99	7	106
SARC	Sarcoma	265	4	269
SKCM	Skin Cutaneous Melanoma	473	2	475
STAD	Stomach adenocarcinoma	394	2	396
TGCT	Testicular Germ Cell Tumors	156	0	156
THCA	Thyroid carcinoma	515	56	571
THYM	Thymoma	124	2	126
UCEC	Uterine Corpus Endometrial Carcinoma	439	45	484
UCS	Uterine Carcinosarcoma	57	0	57
UVM	Uveal Melanoma	80	0	80
Total		8961	727	9688

Supplementary Table S1. List and number of samples and acronyms for cancers analyzed from TCGA.

Supplementary Table S2

ROCs for “HCC-detect” sum and “HCC-spec” + “HCC-detect” sum scores and number of samples with methylation values larger than 0.2

Test Type		ROC	sensitivity	specificity	
HCC detect sum	HCC blood	0.993	95	97	
	HCC other normal	0.948	84	95	
	HCC blood and normal	0.985	91	95	
	HCC NAT	0.919	85	95	
HCC detect +spec sum	HCC blood	0.998	99	99	
	HCC other normal	0.996	95	98	
	HCC blood an normal	0.996	97	98	
	HCC NAT	0.966	92	95	
Number of samples with methylation >0.2					
Sample Group	Vash2	GRID2IP	CHFR	CCNJ	F12
HCC	420	332	388	305	747
Other blood normal no HCC	0	1	5	0	0
	0.587	0.502	0.579	0.442	0.968

Supplementary Table S2. ROC and Methylation Data for HCC Detection

ROCs for “HCC-detect” sum “HCC-spec” + “HCC-detect” sum scores and number of samples with methylation values larger than 0.2.

Supplementary Table S3 HCC Spec Model: Statistical Analysis Summary

Category	Statistic	Value	Intercept	cg14126493
Regression Statistics	Multiple R	0.7987		
	R Square	0.638		
	Adjusted R Square	0.6337		
	Standard Error	0.2572		
	Observations	86		
ANOVA	df (Regression)	1		
	df (Residual)	84		
	SS (Regression)	9.792		
	SS (Residual)	5.557		
	MS (Regression)	9.792		
	MS (Residual)	0.066		
	F	148.034		
	Significance F	0		
Coefficients	Coefficient		0.179	1.347
	Standard Error		0.056	0.111
	t Stat		3.222	12.167
	P-value		0.002	0
	Lower 95%		0.069	1.127
	Upper 95%		0.29	1.567

Supplementary Table S3. Model statistics for "HCC-spec".

The table shows the results of the HCC spec model statistics obtained using the Analysis ToolPak in Microsoft Excel. The table provides information on the regression statistics, including multiple R, R squared, and adjusted R squared, as well as the ANOVA and coefficient values for the intercept and cg14126493. The analysis revealed a significant relationship between cg14126493 and a weighted methylation score for F12 ($F = 148.034$, $p < 0.0001$)

Supplementary Table S4 Statistical significance median HCC-detect M-scores between healthy controls, healthy plasma, CHB, non-HCC cancers and four stages of HCC.

	Control	CHB	Stage A	Stage B	Stage C	Stage D	non-HCC cancers
**Healthy**	ns	ns	***	****	****	****	ns
**Control**		ns	***	****	****	****	ns
**CHB**			*	****	****	****	*
**Stage A**				ns	ns	ns	****
**Stage B**					ns	ns	****
**Stage C**						ns	****
**Stage D**							****

Supplementary Table S4 Median HCC-detect M-score comparison among healthy, CHB, non-HCC cancers, and different stages of HCC

Statistical significance between median HCC-detect M-scores of healthy controls (n=46), healthy plasma (n=50), CHB (n=49), non-HCC cancers (n=102), and four stages of HCC (Stage A+O, n=34, Stage B, n=86, Stage C, n=106 and Stage D, n=76). See Figure 5D.

Supplementary Table S5

Statistical significance median HCC-spec M-scores between between healthy controls, healthy plasma, CHB, non-HCC cancers and four stages of HCC.

	Control	CHB	Stage A	Stage B	Stage C	Stage D	non-HCC cancers
**Healthy**	ns	ns	****	****	****	****	ns
**Control**		ns	****	****	****	****	ns
**CHB**			****	****	****	****	ns
**Stage A**				ns	ns	ns	****
**Stage B**					ns	ns	****
**Stage C**						ns	****
**Stage D**							****

Supplementary Table S5 Statistical significance of HCC-specific methylation scores among different groups and stages.

Statistical significance between median HCC-spec M-scores of healthy controls, healthy plasma, CHB, non-HCC cancers, and four stages of HCC. See Figure 5F.

Supplementary Table S6

Statistical significance of "HCC-detect" predicted probabilities (0 to 1) between the samples from the Dhaka clinical study and healthy plasma from InnovativeTM Research

	Control	CHB	Stage A	Stage B	Stage C	Stage D	non-HCC cancers
**Healthy**	ns	ns	***	****	****	****	ns
**Control**		ns	***	****	****	****	ns
**CHB**			*	****	****	****	*
**Stage A**				ns	ns	ns	****
**Stage B**					ns	ns	****
**Stage C**						ns	****
**Stage D**							****

Supplementary Table S6

Statistical significance of predicted probabilities (0 to 1) between the samples from the Dhaka clinical study and healthy plasma from Innovative^{TM} Research calculated using the logistic regression equation for the "HCC-detect" M scores. See Figure 6C

Supplementary Table S7

Statistical significance "HCC-spec" of sum probabilities scores for the 50 healthy plasma (InnovativeTM Research), healthy controls, chronic hepatitis B 102 non-HCC cancer patients and four stages of HCC.

	Control	CHB	Stage A	Stage B	Stage C	Stage D	non-HCC cancers
**Healthy**	ns	ns	****	****	****	****	ns
**Control**		ns	***	****	****	****	ns
**CHB**			****	****	****	****	ns
**Stage A**				ns	ns	ns	****
**Stage B**					ns	ns	****
**Stage C**						ns	****
**Stage D**							****

Supplementary Table S7 Logistic regression analysis of HCC detection in Dhaka clinical study and healthy plasma**.**

Statistical significance of sum probabilities scores for the 50 healthy plasma (Innovative^{TM} Research), healthy controls, chronic hepatitis B 102 non-HCC cancer patients and four stages of HCC for "HCC-spec" M scores. See Figure 6F

Supplementary Table S8

Statistical significance of predicted probabilities (0 to 1) between the samples from the Dhaka clinical study and healthy plasma from InnovativeTM Research

	Control	CHB	Stage A	Stage B	Stage C	Stage D	non-HCC cancers
**Healthy**	ns	ns	****	****	****	****	ns
**Control**		ns	****	****	****	****	ns
**CHB**			***	****	****	****	ns
**Stage A**				ns	ns	ns	****
**Stage B**					ns	ns	****
**Stage C**						ns	****
**Stage D**							****

Supplementary Table S8 Significance of predicted probabilities for Dhaka clinical study samples vs. healthy plasma using HCC-spec M scores

Statistical significance of predicted probabilities (0 to 1) between the samples from the Dhaka clinical study and healthy plasma from Innovative™ Research calculated using the logistic regression equation for the "HCC-spec" M scores.

Supplementary Table S9 HCC Detect Model: Statistical Analysis Summary

Category	Statistic	Value	Intercept	cg03768777	cg24804544	cg05739190	cg02012576
Regression Statistics	Multiple R	0.811					
	R Square	0.657					
	Adjusted R Square	0.648					
	Standard Error	0.297					
	Observations	145					
ANOVA	df (Regression)	4					
	df (Residual)	140					
	SS (Regression)	23.639					
	SS (Residual)	12.32					
	MS (Regression)	5.91					
	MS (Residual)	0.088					
	F	67.159					
	Significance F	1.3E-31					
Coefficients	Coefficient		0.064	0.751	0.427	0.64	0.804
	Standard Error		0.035	0.159	0.137	0.136	0.18
	t Stat		1.86	4.731	3.116	4.718	4.474
	P-value		0.065	0	0.002	0	0
	Lower 95%		-0.004	0.437	0.156	0.372	0.449
	Upper 95%		0.132	1.065	0.698	0.908	1.16
Model Performance	Sensitivity	0.985					
	Specificity	1					
	Cutoff	0.24					
	Accuracy	0.993					
	AUC	0.991					

Supplementary Table S9. Model statistics for “HCC-detect”.

The table shows the results of the HCC detect model statistics obtained using the Analysis ToolPak in Microsoft Excel. The table provides information on the regression statistics, including multiple R, R squared, and adjusted R squared, as well as the ANOVA and coefficient values for the intercept and four CpG sites (cg03768777, cg24804544, cg05739190, and cg02012576). The analysis revealed a significant relationship between the CpG sites and a weighted methylation score for HCC detection ($F = 67.159$, $p < 0.0001$). In addition, the table includes performance metrics of the HCC detect model, including sensitivity, specificity, cutoff value, accuracy, and AUC.

Supplementary Table S10 Summary of 102 Non-HCC study participants

Cancer Groups	Total N of Enrolled	Stage 1	Stage 2	Stage 3	Stage 4
Bladder Cancer	4	0	1	2	1
Breast Cancer	16	2	7	7	0
Cervical Cancer	12	0	5	6	1
Head and Neck Squamous Cancer	11	0	2	4	5
Lung Cancer	17	0	1	10	6
Colon Cancer	19	3	3	13	0
Esophageal Carcinoma	7	0	0	6	1
Ovarian Cancer	6	1	0	4	1
Prostate Cancer	1	0	1	0	0
Gastric Cancer	5	0	1	2	2
Gall Bladder Cancer	1	0	0	0	1
Renal Cell Carcinoma	1	0	1	0	0
Thyroid Cancer	1	1	0	0	0
Soft Tissue Sarcoma	1	0	0	1	0

Supplementary Table S10. Summary of 102 Non-HCC study participants, presenting the number of enrolled participants in each cancer group and the stage of cancer at the time of enrollment.

Supplementary Table S11 Cross Validation Model Performance Metrics

Training #	R2	RMSE	MAE
1	1	3.25	2.85
2	1	3.25	2.83
3	1	3.24	2.83

*R2- R-Squared
*RMSE is the Root of the Mean of the Square of Errors
*MAE- Mean Absolute Error

Supplementary Table S11. Cross-validation model performance metrics for three training runs. The table shows the R-squared (R2), root mean square error (RMSE), and mean absolute error (MAE) values for each training run. RMSE is calculated as the root of the mean of the square of errors.
