# Supplementary material for: A high-throughput test enables specific detection of hepatocellular carcinoma
Source: Nat Commun. 2023 Jun 7;14:3306. doi: 10.1038/s41467-023-39055-7 (PMC10247794; doi:10.1038/s41467-023-39055-7)
Supplement: Supplementary file 2 — Reporting Summary [file 41467_2023_39055_MOESM2_ESM.pdf]

nature portfolio

Corresponding author(s): David Cheishvili

Last updated by author(s): Apr 14, 2023

Reporting Summary

Nature Portfolio wishes to improve the reproducibility of the work that we publish. This form provides structure for consistency and transparency in reporting. For further information on Nature Portfolio policies, see our Editorial Policies and the Editorial Policy Checklist.

Statistics

For all statistical analyses, confirm that the following items are present in the figure legend, table legend, main text, or Methods section.

n/a	Confirmed	
☐	☒	The exact sample size (n) for each experimental group/condition, given as a discrete number and unit of measurement
☒	☐	A statement on whether measurements were taken from distinct samples or whether the same sample was measured repeatedly
☐	☒	The statistical test(s) used AND whether they are one- or two-sided<br>*Only common tests should be described solely by name; describe more complex techniques in the Methods section.*
☒	☐	A description of all covariates tested
☐	☒	A description of any assumptions or corrections, such as tests of normality and adjustment for multiple comparisons
☐	☒	A full description of the statistical parameters including central tendency (e.g. means) or other basic estimates (e.g. regression coefficient) AND variation (e.g. standard deviation) or associated estimates of uncertainty (e.g. confidence intervals)
☐	☒	For null hypothesis testing, the test statistic (e.g. F, t, r) with confidence intervals, effect sizes, degrees of freedom and P value noted<br>*Give P values as exact values whenever suitable.*
☒	☐	For Bayesian analysis, information on the choice of priors and Markov chain Monte Carlo settings
☒	☐	For hierarchical and complex designs, identification of the appropriate level for tests and full reporting of outcomes
☒	☐	Estimates of effect sizes (e.g. Cohen's d, Pearson's r), indicating how they were calculated

Our web collection on statistics for biologists contains articles on many of the points above.

Software and code

Policy information about availability of computer code

Data collection	The data was obtained from open sources, specifically TCGA and NCBI with accession codes are mentioned in Table 1 of the manuscript. To obtain the TCGA data used in this study, the Genomic Data Commons (GDC) Data Transfer Tool (DTT) client version 1.6.1. was utilized.
Data analysis	To process DNA methylation, we utilized Bismark version 0.23.0 and trim_galore version 0.6.6. For statistical analysis and data visualization, we employed Prism. In addition, we used GENE E for visualization purposes.

For manuscripts utilizing custom algorithms or software that are central to the research but not yet described in published literature, software must be made available to editors and reviewers. We strongly encourage code deposition in a community repository (e.g. GitHub). See the Nature Portfolio guidelines for submitting code & software for further information.

Data

Policy information about availability of data

All manuscripts must include a data availability statement. This statement should provide the following information, where applicable:

- Accession codes, unique identifiers, or web links for publicly available datasets
- A description of any restrictions on data availability
- For clinical datasets or third party data, please ensure that the statement adheres to our policy

The data that support the findings of this study are available from the corresponding author upon reasonable request. We are willing to share the data with other researchers for non-commercial research purposes upon reasonable request and the signing of an MTA agreement with our institution.

Human research participants

Policy information about studies involving human research participants and Sex and Gender in Research.

Reporting on sex and gender	The study did not consider sex and gender in the study design. However, sex information was collected during checkups and questionnaires that patients filled out during their visit, and by providing informed consent. Demographic information about sex distribution in the study is described in Table 2. Although sex and gender were not considered relevant to the study as the focus was on studying HCC oncomarkers that are applicable to both sexes, sex was still used as a potential covariate.
Population characteristics	402 participants were recruited from the Dhaka area to the study included 49 healthy controls, 51 Chronic hepatitis B patients, 102 non-HCC patients and 302 HCC patients from stages 0 to D (HCC 0 n=2, HCCA n=32, HCC B n=86, HCC C n=106, HCC D n=76. We obtained covariates information from the participants regarding their age, sex, alcohol consumption, and smoking status. The sex distribution was found to be similar across all groups. However, the HCC groups exhibited a somewhat significant lower prevalence of alcohol use and a higher proportion of smokers compared to the other groups. This is summarized in Table 2 of the manuscript.
Recruitment	The study participants were recruited from the International Centre for Diarrhoeal Disease Research, Bangladesh (ICDDR,B) and did not self-select. As the participants were coming to the hospital and not self-selected, there was not self-selection bias in the study. One potential bias that may be present is selection bias, where the participants who come to the hospital may not be representative of the broader population. For example, they may be sicker or have more severe symptoms, which could impact the results of the study.
Ethics oversight	Study protocol was approved by IRB board of icddr,b (Dhaka, Bangladesh)

Note that full information on the approval of the study protocol must also be provided in the manuscript.

Field-specific reporting

Please select the one below that is the best fit for your research. If you are not sure, read the appropriate sections before making your selection.

☒ Life sciences ☐ Behavioural & social sciences ☐ Ecological, evolutionary & environmental sciences

For a reference copy of the document with all sections, see nature.com/documents/nr-reporting-summary-flat.pdf

Life sciences study design

All studies must disclose on these points even when the disclosure is negative.

Sample size	To estimate the minimum sample size required to validate our findings in a new clinical study we performed power analyses. A power calculation using the pooled standard deviation of the methylation scores (sigma) for the healthy blood and HCC tissues (0.31) and desired power of 0.8 shows that a sample size of 40 for each group is required to detect a delta beta of 0.2 between cancer and control. We also performed a power calculation of the cfDNA plasma methylation data from GSE63775 study on each gene region separately. A power calculation using the pooled standard deviation (spooled=71.01) of average reads for all 5 genes: CCNJ, F12, VASH2, GRID2IP and CHFR in normal and HCC plasma and desired power of 0.8 shows that a samples size of only 9 per group was sufficient to detect difference of 98 reads between the groups. We reasoned that a samples size of 400 would be sufficiently powered to detect significant differences in methylation between HCC and control.
Data exclusions	Exclusion criteria were unwilling or unable to provide informed consent, unwilling or unable to comply with requirements of protocol, participation in a different clinical trial investigating a vaccine, drug, medical device or medicinal procedure less than 4 weeks preceding the current study, planned participation in another clinical trial during present study period, known case of cirrhosis, any other known inflammatory disease (bacterial or viral infection with the exception of hepatitis B or C), known case of diabetes, asthma, autoimmune disease, any other diagnosed cancer, for healthy controls any known inflammatory or infectious disease including Hepatitis B and Hepatitis C and any diagnosis of chronic disease, cancer medication use or drugs of abuse.
Replication	The experimental findings were replicated in 21 different datasets, which are summarized in Table 1. Additionally, we were able to successfully replicate the bioinformatic analysis of the FASTQ file from the clinical study of the Bangladesh cohort.
Randomization	Participants were allocated into experimental groups by clinical and pathologists who diagnosed the cancer. The allocation was based on the participants' cancer and/or healthy status, with those diagnosed with cancer assigned to one group and those with no cancer diagnosis assigned to another.
Blinding	Blood sample collection ad plasma separation was performed at icddr,b in Dhaka Bangladesh and was then shipped to HKG epitherapeutics for further analysis. The HKG epitherapeutics lab team was blinded on the identity of the samples throughout the lab analytic procedures. Data was then analyzed in Montreal and shared with icddr,b who provided the results to the respective clinical personnel.

Reporting for specific materials, systems and methods

We require information from authors about some types of materials, experimental systems and methods used in many studies. Here, indicate whether each material, system or method listed is relevant to your study. If you are not sure if a list item applies to your research, read the appropriate section before selecting a response.

Materials & experimental systems

n/a	Involved in the study
☒	☐ Antibodies
☒	☐ Eukaryotic cell lines
☒	☐ Palaeontology and archaeology
☒	☐ Animals and other organisms
☐	☒ Clinical data
☒	☐ Dual use research of concern

Methods

n/a	Involved in the study
☒	☐ ChIP-seq
☒	☐ Flow cytometry
☒	☐ MRI-based neuroimaging

Clinical data

Policy information about clinical studies

All manuscripts should comply with the ICMJEguidelines for publication of clinical research and a completedCONSORT checklist must be included with all submissions.

Clinical trial registration: NCT03483922

Study protocol: The study protocol can be obtained from the corresponding author upon reasonable request.

Data collection: The data were collected at the International Centre for Diarrhoeal Disease Research, Bangladesh (ICDDR,B) located in Dhaka, Bangladesh. Recruitment and data collection took place between 2018 and December 2021.

Outcomes: The primary outcome measure was the calculation of M Scores and HCC Probability Scores. These measures were pre-defined to detect cancer broadly, regardless of stage. The median values of the percentage methylation for each gene, ranging from 0 to 100, were normalized using log2 transformation. An "HCC-detect" M score was then computed by adding the normalized medians of CHFR, VASH2, CCNJ, and GRID2IP regions, which were given equal weight. Similarly, an "HCC-spec" M score was computed based on the median methylation of the F12 region, which correctly classifies HCC samples from tumors of different cell-type origins.

We used logistic regression in Prism to model the M score as a predictor of the probability of HCC and computed a predicted probability for each person using the logistic regression equation. These measures were assessed by computing the averages of the sum of probabilities per group, which are presented in the outcome measure data table.
